# Chemistry and biology of specialized metabolites produced by *Actinomadura*

**DOI:** 10.1039/d3np00047h

**Published:** 2023-12-15

**Authors:** Yousef Dashti, Jeff Errington

**Affiliations:** a Faculty of Medicine and Health, University of Sydney Sydney NSW 2015 Australia yousef.dashti@sydney.edu.au

## Abstract

Covering: up to the end of 2022

In recent years rare Actinobacteria have become increasingly recognised as a rich source of novel bioactive metabolites. *Actinomadura* are Gram-positive bacteria that occupy a wide range of ecological niches. This review highlights about 230 secondary metabolites produced by *Actinomadura* spp., reported until the end of 2022, including their bioactivities and selected biosynthetic pathways. Notably, the bioactive compounds produced by *Actinomadura* spp. demonstrate a wide range of activities, including antimicrobial, antitumor and anticoccidial effects, highlighting their potential in various fields.

## Abstract 2

*Actinomadura* represents a promising source of natural products. This review emphasizes the specialized metabolites produced by this genus, their biological activities, and selected biosynthetic pathways.

## Graphical abstract

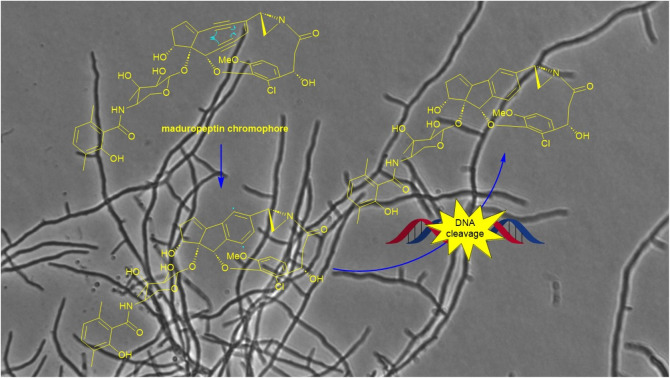

## 1. Introduction

Actinobacteria (organisms belonging to the phylum Actinomycetota) are a diverse group of bacteria that are well-known for their potential to produce natural products with diverse agricultural and industrial applications, as well as for their potential to serve as sources of novel medicines.^1,2^ Among them, the genus *Streptomyces* has been extensively studied for its specialized metabolites, which have yielded about two-thirds of all antibiotics and several anticancer drugs.^3^ However, research into this genus over the last four decades has mostly resulted in the rediscovery of known molecules.

While traditional culture and screening approach failed to provide novel chemical entities, antimicrobial resistance among pathogens has become a major concern, urging the need for new antibiotics with a different mode of action.^4,5^ To address this issue, two main strategies have been implemented in the field of microbial natural product (NP) discovery. The first involves accessing the products of cryptic biosynthetic gene clusters (BGCs), while the second strategy focuses on understudied Actinobacteria. Rapid advances in next-generation sequencing technology, in conjunction with our understanding of the molecular logic for the assembly of microbial NPs, has revealed that the products of less than 10% of the BGCs encoded in the genome of well-studied strains are known.^6,7^ To access the products of cryptic BGCs, various genetic-dependent and independent methods have been used, and both methods have led to the discovery of many new NPs.^7^ The approach based on understudied Actinobacteria mainly relies on new isolation methods. Recent advances in isolation methods have increased efforts to culture “hard-to-grow” strains of bacteria, leading to a higher proportion of novel metabolites being identified from rare actinomycetes, such as *Actinomadura*.^8,9^

*Actinomadura* is a Gram-positive genus of Actinobacteria belonging to the Thermomonosporaceae family. These bacteria are ubiquitous and are capable of thriving in diverse ecological niches, including marine and terrestrial environments. Some species of *Actinomadura* are opportunistic human pathogens.^10^ In this review, we present a comprehensive overview of the secondary metabolites produced by *Actinomadura*, their bioactivities, and selected biosynthetic pathways. The 230 or so bioactive compounds produced by *Actinomadura* exhibit a broad range of activities and their biosynthetic routes are diverse. The diversity of *Actinomadura* and their potential to produce novel bioactive compounds make them an attractive target for drug discovery research.

## 2. Polyketides

### 2.1 Polyethers

Polyether ionophores are a group of biologically active natural products with the ability to form complexes with metal cations and transport them across the lipid bilayer of cell membranes. This process disrupts the Na^+^/K^+^ gradient and consequently increases the osmotic pressure within the cell, leading to swelling, vacuolization, and finally cell death.^11–15^ Due to differences in structural features of the bacterial cell wall, polyether ionophores are mainly active against Gram-positive strains. The porous peptidoglycan layer of Gram-positive bacteria allows small molecules to pass through and reach the cytoplasmic membrane, where the lipophilic ionophore-metal complex can pass through.^16^ In addition to their antibacterial properties, polyether ionophores are highly effective against coccidian protozoa, parasites that cause coccidiosis in wild and domestic animals, resulting in damage to the intestinal tract. Infected animals have stunted growth due to nutrient malabsorption and dehydration and are susceptible to other infections. Some of the polyether's from *Actinomadura* are commercially used as anticoccidial agents in animal food.^16^

**Fig. 1:**
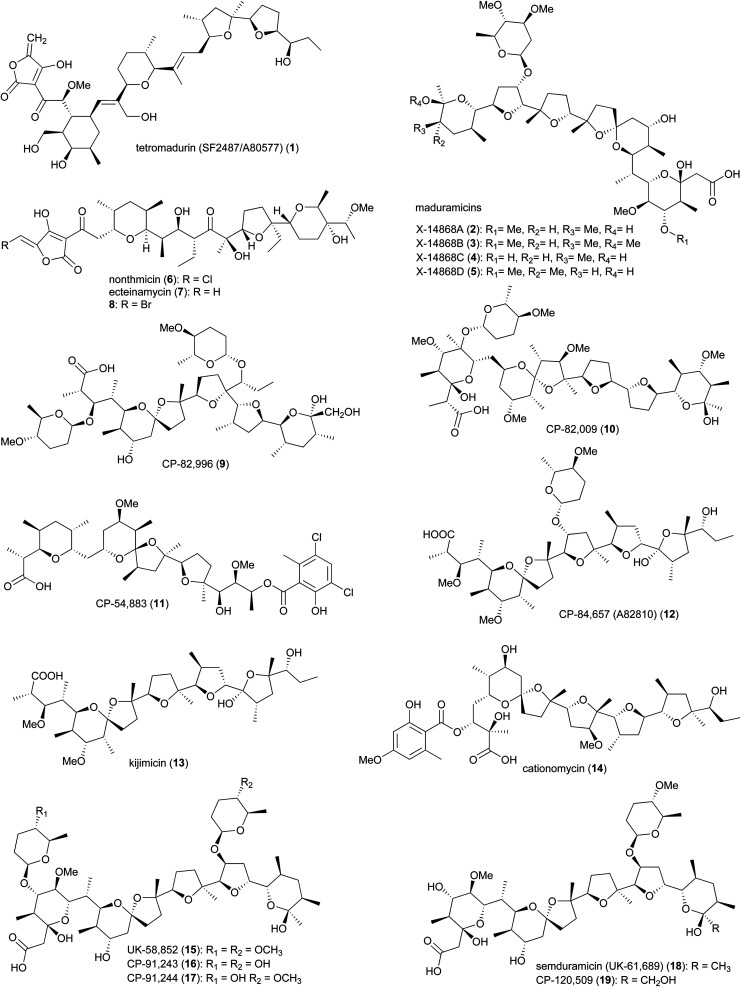
Structures of polyethers produced by *Actinomadura*.

The polyether tetronate ionophore tetromadurin (1) (originally known as SF2487 and A80577) is produced by *Actinomadura verrucosospora* (Fig. 1).^17,18^ Like other ionophore antibiotics, tetromadurin disrupts ion signalling across cell membranes.^17^ In addition to antibacterial activity, the compound also exhibited activity against influenza virus and *Plasmodium falciparum*.^17,19^

**Fig. 2:**
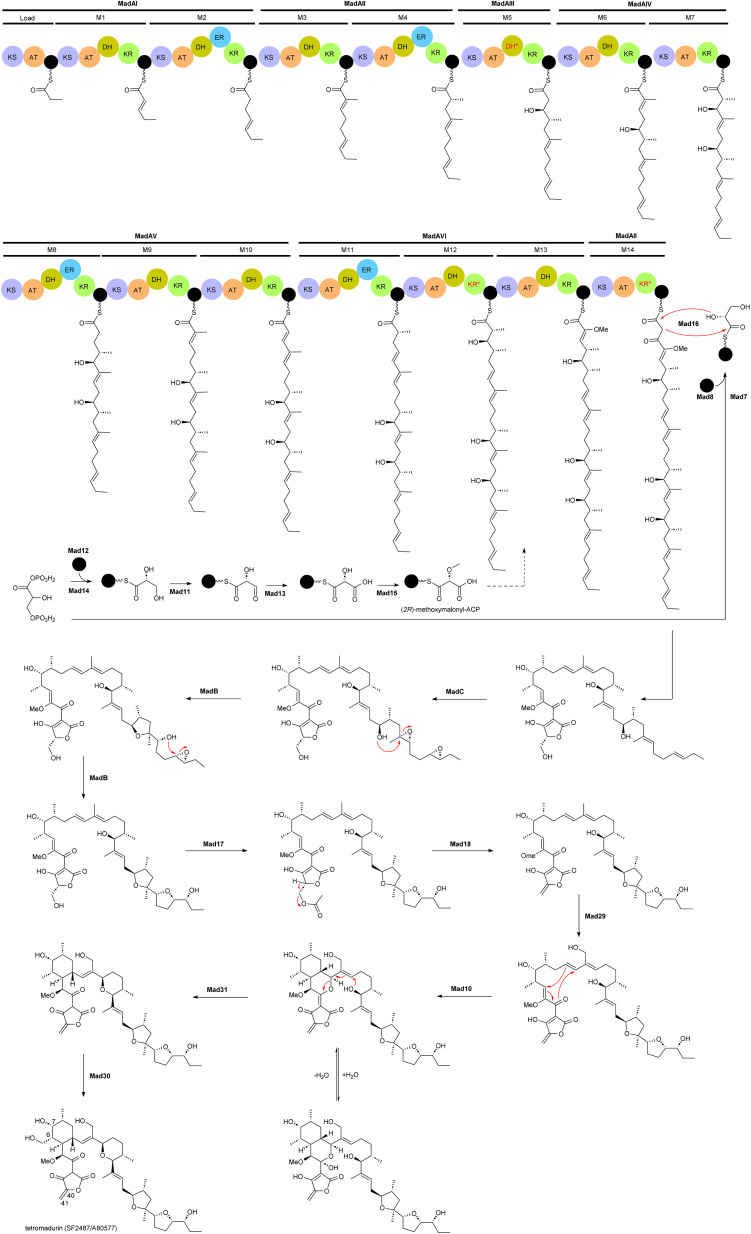
Proposed biosynthetic pathway for tetramadurin, a 31-carbon polyketide skeleton, by the *mad* gene cluster in *A. verrucosopora*, based on the bioinformatics analysis and functional analysis of gene deletions.^20^

The biosynthetic gene cluster of tetramadurin, *mad*, was identified from the sequenced genome of *A. verrucosospora*.^20^ The biosynthetic pathway for assembly of the final products proposed based on bioinformatics analysis and gene deletion studies is shown in Fig. 2. The *mad* gene cluster contains seven genes (*madAI*–*madVII*) encoding type I polyketide synthases incorporating one loading module and fourteen extension modules. The latter recruit six malonyl-CoA, eight (*2S*)-methylmalonyl-CoA and an uncommon (*2R*)-methoxymalonyl-ACP units for assembly of the 31-carbon polyketide skeleton of tetramadurin. Five genes, *mad11*–*mad15*, encode proteins responsible for biosynthesis of the unusual (*2R*)-methoxymalonyl-ACP from 1,3-bisphosphoglycerate, which is then activated by the AT domain of module 13. Chain release is catalysed by the concerted action of Mad7, Mad8 and Mad16. *mad8* encodes a standalone acyl carrier protein (ACP) that serves as a scaffold for catalytic formation of glycerol-ACP by Mad7. The tetronate formation that leads to chain release is catalysed by the FabH-like protein Mad16. Acetyltransferase Mad17 and dehydratase Mad18, together, are proposed to be responsible for exocyclic double bond formation on the tetronate ring. Tetrahydrofuran rings are formed by an epoxidase, MadC, followed by epoxide hydrolase, MadB. Two cyclases, Mad10 and Mad31, are proposed to produce the cyclohexane and tetrahydropyran rings. Finally, the methyl group at the C6 position is hydroxylated by Mad30 to yield the final product.^20^

An unusual feature of *mad* gene cluster is the KR module 12. Sequence analysis showed that this module lacks the key catalytic tyrosine residue and is also missing the NADPH binding site, likely making the module inactive. However, these observations are not consistent with the hydroxyl group at position 7 in the final structure. In addition, module 12 also contains an active DH domain, which is expected to generate a C6–C7 double bond. The process leading to formation of the hydroxyl group at C7 is therefore unclear.^20^

Maduramicin X-14868A (2) and minor analogues 3–5 were first isolated from *A. yumaensis* (formerly *Nocardia* strain X-14868).^21^ Due to its higher efficacy and lower toxicity maduramicin X-14868A possesses the largest market share among anti-coccidiosis polyether antibiotics in the poultry sector. In addition to anticoccidial activity, the compound also has anti-malarial and anticryptosporidial activity. The type 1 polyketide synthase (T1PKS) gene cluster of maduramicin, *mad* (note that the biosynthetic gene clusters of tetramadurin and madurastatin, described later, are also annotated as *mad*), was identified *via* whole genome sequencing of *Actinomadura* strain J1-007, the maduramicin producing industrial strain. Overexpression of the type II thioesterase MedTE within the *mad* gene cluster led to a 30% increase in maduramicin production.^22^

The polyether polyketide nonthmicin (6) and its dechloro congener ecteinamycin (7) were isolated from a soil-derived *Actinomadura* strain K4S16.^23^ Both compounds demonstrated potent antiinvasive activity against murine carcinoma colon 26-LS cells with IC_50_ values of 0.017 and 0.15 μM, respectively. They also showed neuroprotective properties and potent antimicrobial activity against Gram-positive bacteria.^23^ The initial isolation of ecteinamycin was from a marine *Actinomadura* strain isolated from an ascidian. In that study, 7 displayed potent activity against a toxigenic strain of *Clostridioides difficile* NAP1/B1/027, with an MIC of 59 ng μL^−1^.^24^ Mode of action studies suggested the activity of ecteinamycin against *C. difficile* is due to its ionophore properties which interfere with bacterial ion transport.^24^ The broad range of apparent activities are consistent with a relatively non-specific toxicity, consistent with an ionophore mode of action.

The biosynthetic pathways for nonthmicin and ecteinamycin were predicted based on bioinformatic analysis of the genome of *Actinomadura* strain K4S16.^25^ The type-I PKS gene cluster *t1pks-4* encodes six PKS enzymes containing 12 modules, along with auxiliary genes encoding the enzymes involved in formation of tetronic acid moiety, as well as epoxidase and epoxide hydrolase/cyclase's. Together, these are suggested to assemble compounds 6 and 7. Substrate specificities of the AT domains were shown to be consistent with the structure. This was further confirmed by a feeding experiment, adding labelled precursor to the culture medium.^25^ A predicted halogenase K4S16_09_00450 in the gene cluster, suggested to be responsible for chlorination, was found to be active for bromine as well, as addition of sodium bromide to the culture medium resulted in the replacement of chlorine, yielding a brominated congener 8.^25^

In addition to the above polyether ionophores, several other very potent antimicrobial and anticoccidial polyethers are produced by *Actinomadura* species, including CP-82,996 (9) isolated from the fermentation broth of *Actinomadura* strain ATCC 53764,^26^ CP-82,009 (10) from *Actinomadura* strain ATCC 53676,^27,28^ CP-54,883 (11) from *A. routienii*.^29,30^ CP-84,657 (A82810) (12) from *Actinomadura* strain ATCC 53708 (*A. fibrosa*).^31,32^ and kijimicin (13) from *Actinomadura* strain MI215-NF3.^33^ Kijimicin was also reported to inhibit the replication of human immunodeficiency virus.^34,35^ Cationomycin (14), was purified from a fermentation broth of *A. azurea*.^36,37^ The aromatic side fragment of cationomycin was found to be important for its biological activity as removing this aromatic ring reduced the activity.^38,39^ The di-glycosylated polyether UK-58,852 (15) produced by *A. roseorufa* ATCC 53666. Chemical mutagenesis of this strain resulted in production of desmethyl congener antibiotics CP-91,243 (16) and CP-91,244 (17), as well as mono-glycosylated semduramicin (UK-61,689) (18) and CP-120,509 (19).^40–43^

### 2.2 Spirotetronates

**Fig. 3:**
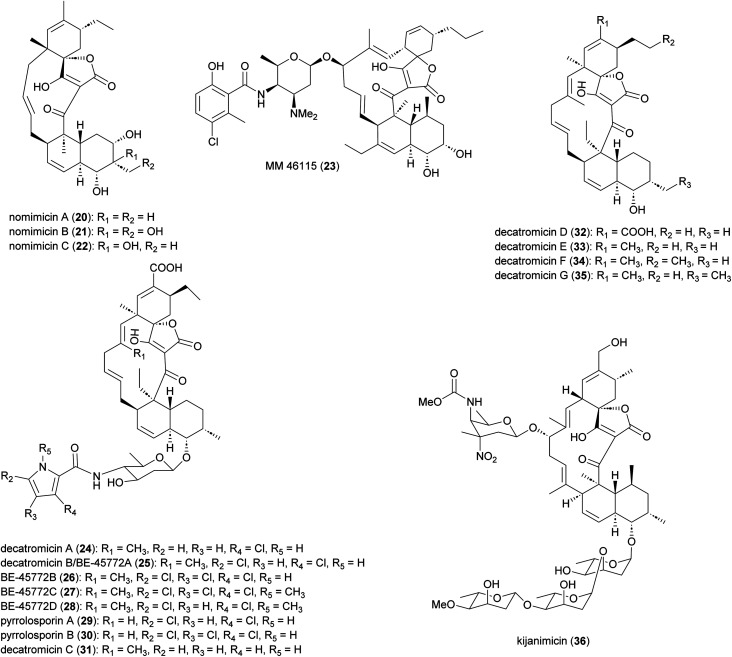
Structure of spirotetronate polyketides.

Spirotetronate polyketides are microbial metabolites with intriguing chemical structures and bioactivities. These metabolites are identified by the presence of a tetronic acid moiety spiro-linked to a cyclohexene ring, that is embedded in a macrocycle.^44^ All spirotetronates so far identified from *Actinomadura* (Fig. 3) are categorized as class II based on their *trans*-decalin ring.^45^ The spirotetronate nomimicin A (20), isolated from the culture broth of *Actinomadura* strain TP-A0878, showed antibacterial activity against *Micrococcus luteus, Candida albicans* and *Kluyveromyces fragilis*, with MIC values in the range of 6.3 to 12.5 μg mL^−1^.^46^ The same compound was isolated from *Actinomadura* strain AKA43, along with nomimicins B (21), C (22) and the precursor metabolite nomimicin D. Nomimicins B and D inhibited the growth of *Kocuria rhizophila* and *Bacillus subtilis*, in the MIC range of 6.5 to 12.5 μg mL^−1^, but were inactive against *C. albicans*. Additionally, nomimicins B and C demonstrated cytotoxicity against P388 murine leukemia cells with IC_50_'s of 33 and 89 μM, accordingly.^47^

Several *A. pelletieri* strains were shown to produce the chlorinated macrolide MM 46115 (23), which is active against parainfluenza virus 1 and 2.^48^ Spirotetronate decatromicins A (24) and B (25) produced by *Actinomadura* strain MK73-NF4 are potent growth inhibitors of *Staphylococcus aureus*, *B. cereus* and *B. subtilis*.^49,50^ Congener metabolites BE-45722B (26) and BE-45722C (27), along with decatromicin B (BE-45722A), were isolated from culture extracts of *Actinomadura* strain 2EPS, and also had potent activity against several Gram-positive bacteria. In addition, 25–27 were found to have significant activity against *C. perfringens* and *C. difficile*, with MICs comparable to that of vancomycin.^51^ Additional analogues of this spirotetronate compound series (28–35), along with those previously reported (24–27), were isolated from culture extracts of *Actinomadura* strain A30804.^52^ Structure–activity relationship studies of this set of compounds suggested that the presence of at least two chloro-substitutions on the pyrrole ring is important for antibacterial activity.^52^

**Fig. 4:**
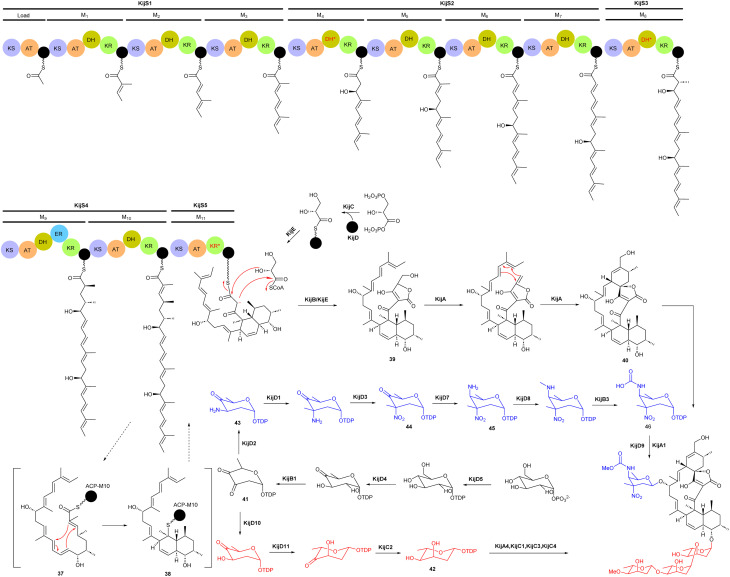
Biosynthetic pathway for the kijanimicin aglycone core, involving PKS genes, glycerol-derived three-carbon units, and intramolecular cyclizations, leading to formation of the octahydronaphthalene and spirotetronate rings. Two different sugar biosynthetic pathways bifurcate from intermediate TDP-2,6-dideoxy-3,4-diketo-d-glucose (41).

Spirotetronate kijanimicin (36), produced by *A. kijaniata*, has a broad spectrum of antimicrobial activity against several Gram-positive bacteria, anaerobes, *P. falciparum*, and also exhibited antitumor activity in a mouse lymphatic leukaemia model.^53–57^ The aglycone of kijanimicin is decorated with three l-digitoxose moieties and a rare nitro sugar, d-kijanose. The pentacyclic core is assembled by the combined action of a modular type I polyketide synthase, incorporation of a three carbon units derived from glycerol, and two intramolecular cyclizations, leading to formation of the octahydronaphthalene and spirotetronate rings (Fig. 4).^58^ Five PKS genes in the *kij* cluster encode PKS proteins KijS1-KijS5, which incorporate a loading module and eleven extender modules: these are responsible for construction of an ACP-11 bound PKS intermediate. There are two further steps in maturation of the kijanimicin aglycone core. A “Diels–Alder-type” intramolecular cyclization between the dienophile and diene of intermediate 37 forms the intermediate 38 with an octahydronaphthalene ring. It is suggested that this cyclization could happen when the polyketide intermediate is tethered to the ACP module 10.^58^ Five enzymes KijABCDE are proposed to activate the glycerol-derived three-carbon unit, attach it to the polyketide intermediate, and subsequently cyclize to generate the spirotetronate ring. It is proposed that KijC uses d-1,3-bisphosphoglycerate as a substrate to generate d-glycerate and load it onto the stand-alone ACP, KijD. The N-terminal acyltransferase domain of KijE then forms glyceryl-CoA by transfer of glycerol from glycerol-*S*-KijD to coenzyme A. KijB then condenses the glyceryl-CoA to the polyketide intermediate tethered to ACP-11, followed by lactonization catalysed by the C-terminal thioesterase domain of KijE. Finally, the polyketide is released from the PKS assembly line. The flavin-dependent monooxygenase/oxidoreductase KijA is suggested to catalyse the dehydration of 39 leading to intramolecular cyclization to form the mature aglycone, 32-deoxy kijanolide (40).^58^

Biosynthesis of both l-digitoxose and d-kijanose begins with the activities of d-glucose-1-phosphate thymidylyltransferase KijD5 and TDP-d-glucose 4,6-dehydratase KijD4, which catalyse the conversion of glucose-1-phosphate to TDP-4-keto-6-deoxy-d-glucose. The 2,3-dehydratase KijB1 then catalyses the formation of TDP-2,6-dideoxy-3,4-diketo-d-glucose (41). At this stage, the biosynthesis of the two sugars diverges into separate pathways. In the TDP-l-digitoxose pathway, KijD10 reduces C-3 to yield TDP-4-keto-2,6-dideoxy-d-glucose, followed by the action of epimerase KijD11 and 4-reductase KijC2 to complete the formation of TDP-l-digitoxose (42). In the d-kijanose pathway, the aminotransferase KijD2 converts 41 to 43, which is then *C*-methylated by methyltransferase KijD1. Flavoprotein KijD3 is proposed to catalyse oxidation of the 3-amino group to form 44. The C-4 aminotransferase KijD7 is postulated to install the second amino group, converting 44 to 45. Intermediate 45 is further modified to form a methylcarbamate moiety *via* the collaborative action of three enzymes, either before or after sugar attachment to the aglycone. The *N*-methyltransferase KijD8 is predicted to methylate the C-4 amine. The flavin-dependent oxidoreductase KijB3 then oxidizes the methyl group to form a carboxylate that is methylated by the *O*-methyltransferase KijA1, forming the final, highly functionalized d-kijanose (46). Four glycosyltransferases identified in the *kij* gene cluster are proposed to install these sugars on the 32-deoxy kijanolide aglycone core.^58^

### 2.3 Fluvirucins

**Fig. 5:**
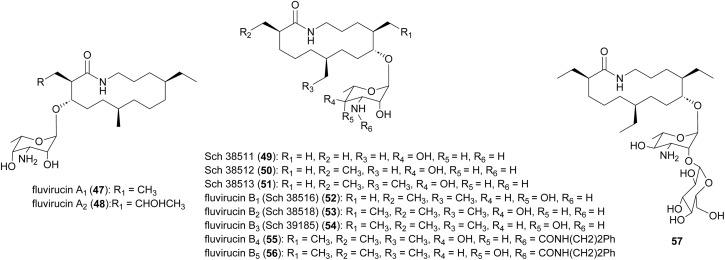
Structure of fluvirucins.

Macrolactam fluvirucins (47–57) were isolated from several *Actinomadura* strains (Fig. 5). Some analogues exhibited potent inhibitory activity against influenza virus, *S. aureus*, *Bacteroides fragilis*, *C. albicans*, and *Cryptococcus neoformans*.^59–65^

**Fig. 6:**
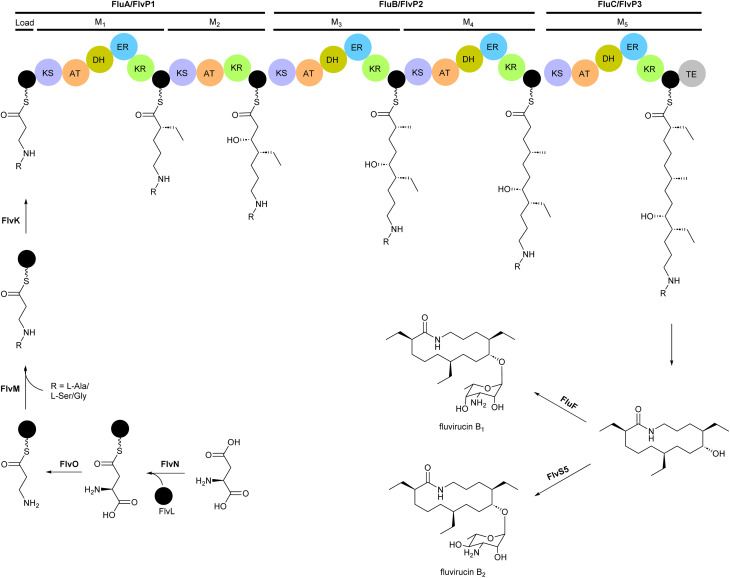
Modular type I PKS gene clusters of fluvirucins B1 and B2 from *A. vulgaris* and *A. fluva* subsp. indica, respectively. Feeding experiment shows incorporation of labelled l-aspartic acid and biochemical studies propose a pathway for the generation and loading of β-alanine, a unique component of the macrolactam structure.

The modular type I PKS gene clusters of fluvirucins B_1_ (*flu*) and B_2_ (*flv*) from *A. vulgaris* and *A. fluva* subsp. *indica*, respectively, comprises three PKS genes encoding five extender modules flanked by an N-terminal loading ACP and a C-terminal TE (Fig. 6).^66,67^ Except module 2, all extender modules contain full sets of tailoring domains KR, DH and ER. Fluvirucin macrolactams contain an unusual β-amino acid unit as part of their core macrolide structure.^66,67^ Feeding experiment showed ^15^N incorporation from labelled l-aspartic acid suggesting that β-alanine moiety originates from l-aspartate *via* decarboxylation of the α-carboxyl group.^68^ Biochemical studies proposed the following pathway for generation and loading of β-alanine. First l-aspartate activated by adenylation enzyme FlvN and ligated to FlvL, a stand-alone acyl carrier protein (ACP), followed by α-decarboxylation of aspartyl-FlvL by the pyridoxal-phosphate (PLP)-dependent decarboxylase FlvO. The adenylation enzyme FlvM then aminoacylates the β-alanyl-FlvL intermediate to give dipeptidyl-FlvL, which is then transferred to the loading ACP domain of FlvP1 by an amino acyltransferase (AT) FlvK.^67,69^ Several of these enzymes demonstrated substrate promiscuity. FlvM was shown to aminoacylate β-alanyl-FlvL with l-alanine, l-serine, and glycine, and FlvK could transfer all three of these dipeptidyl-FlvL moieties to the loading ACP of FlvP1.^69^ Following macrolactamization and release of the product from the PKS assembly line by TE, glycosyltransferases FluF and FlvS5 attach an amino sugar to the polyketide backbone to produce the final fluvirucins B_1_ and B_2_, respectively.

### 2.4 Enediynes

**Fig. 7:**
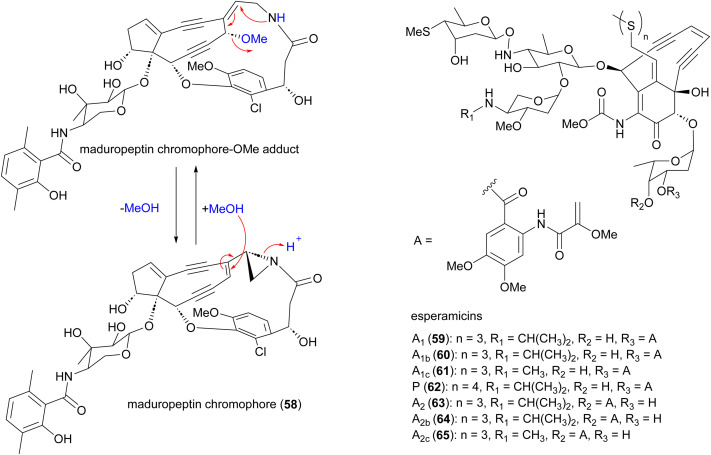
Structure of maduropeptin, maduropeptin-methanol adduct and esperamicins enediynes.

Enediynes are characterized by a shared 1,5-diyne-3-ene motif within a nine- or ten-membered carbocyclic core.^70–72^ The antibiotic enediynes maduropeptin (58)^73–75^ and esperamicins (59–65)^76–79^ were isolated from *A. madurae* ATCC 39144 and *A. verrucosospora*, respectively (Fig. 7). Maduropeptin consists of a noncovalent complex of a nine-membered enediyne chromophore (58) and an apoprotein. Activation requires denaturation of the protein and release of the chromophore to yield a methanol adduct, through nucleophilic addition of methanol to the double bond of the enediyne.^75^ This methanol adduct is more stable but can regenerate the parent chromophore.^75^ Total synthesis of the maduropeptin chromophore led to structural revision of the amino sugar moiety.^80^

**Fig. 8:**
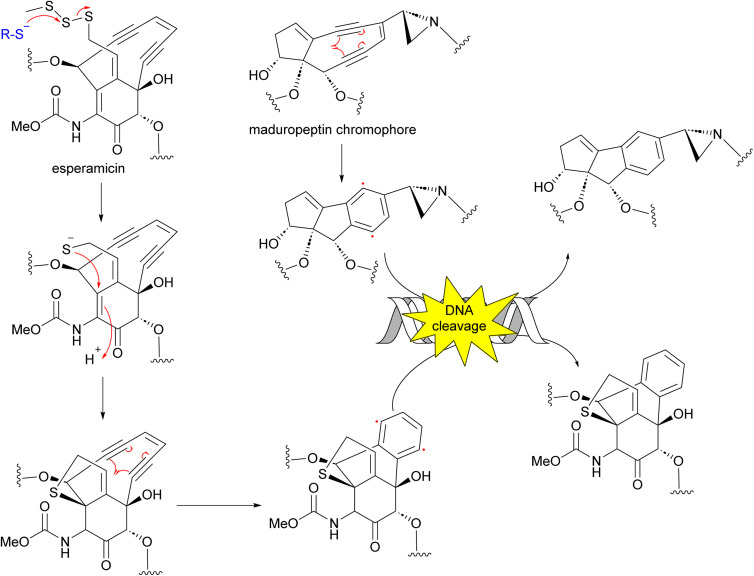
Mechanism of action of enediynes in antitumor and antibacterial activity through Bergman cyclization and DNA cleavage.

Enediynes have been shown to have limited anti-tumour activity in mouse^73^ but they are highly toxic due to their ability to break DNA. Upon an environmental trigger such as UV or thiol activation, the enediyne core undergoes Bergman cyclization to form a benzenoid diradical. The maduropeptin chromophore is exceptionally reactive and without activation undergoes Bergman rearrangement. To complete the aromaticity of the benzene ring, the diradical species abstracts two hydrogens from DNA. This event leads to DNA cleavage and potentially, cell death (Fig. 8).^81,82^

**Fig. 9:**
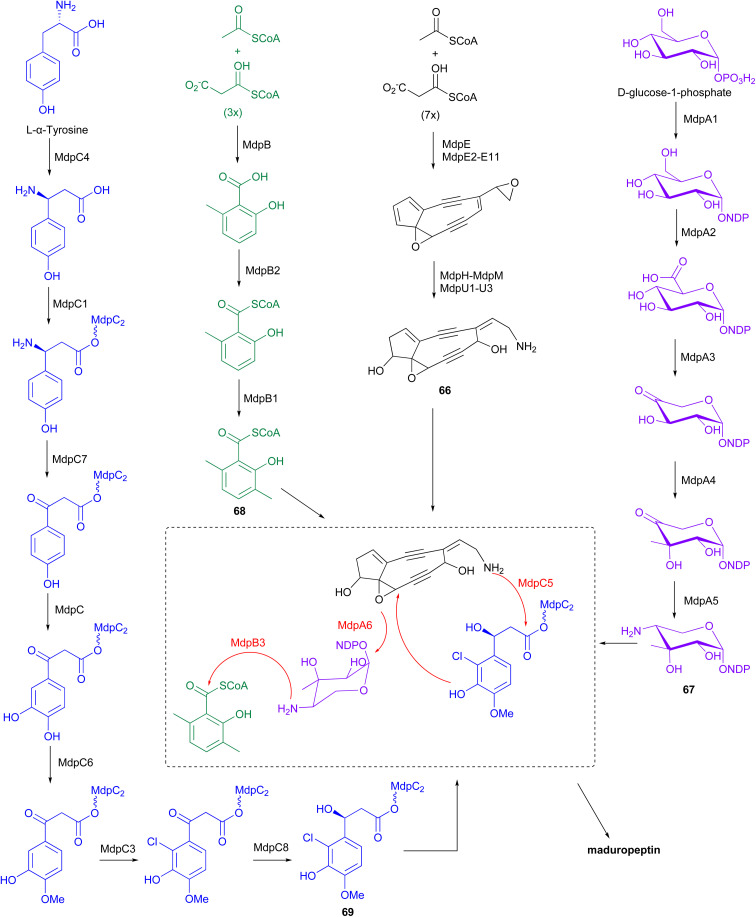
The biosynthetic pathway of maduropeptin chromophore involves the iterative type I PKS MdpE and several tailoring enzymes. These enzymes facilitate modifications, including the addition of an amino sugar, 3,6-dimethylsalicylyl-CoA, and (*S*)-3-(2-chloro-3-hydroxy-4-methoxyphenyl)-3-hydroxypropionic acid, which are attached to the enediyne core intermediate.

The BGC for maduropeptin, *mdp*, was cloned from *A. madurae* ATCC 39144.^83^ The enediyne core of the maduropeptin chromophore (66) is assembled by the iterative type I PKS MdpE and then modified by several tailoring enzymes (Fig. 9). The *mdp* cluster also contains genes encoding enzymes for biosynthesis and attachment of an amino sugar 67 (*mdpA*–*mdpA6*), 3,6-dimethylsalisylyl-CoA 68 (*mdpB*–*mdpB3*), and (*S*)-3-(2-chloro-3-hydroxy-4-methoxyphenyl)-3-hydroxypropionic acid 69 (*mdpC*–*mdpC8*) to produce the final maduropeptin chromophore. Biosynthesis of the amino sugar begins with formation of TDP-glucose from d-glucose-1-phosphate by the enzyme α-d-glucopyranosyl-1-phosphate thymidyltransferase, MdpA1. TDP-glucose is then converted to TDP-glucuronic acid by the dehydrogenase MdpA2. Formation of thymidyl 5′-β-*l*-threo-pentapyranosyl-4′′-ulose diphosphate from TDP-glucuronic acid, catalysed by the decarboxylase MdpA3, followed by incorporation of a methyl group by the C-methyltransferase, MdpA4. Transamination by MdpA5 generates TDP-4-amino-4-deoxy-3-methyl-β-d-ribose 67, which is then attached to the enediyne core intermediate by the glycosyltransferase MdpA6.^83^

The iterative type I PKS, MdpB, is proposed to be responsible for the production of 6-methylsalicylic acid. This product is then activated by the CoA ligase, MdpB2, and methylated by the C-methyltransferase, MdpB1, to form the 3,6-dimethylsalicylyl-CoA thioester 68, before coupling to the enediyne core by acyltransferase, MdpB3.^83,84^ Biosynthesis of (*S*)-3-(2-chloro-3-hydroxy-4-methoxyphenyl)-3-hydroxypropionic acid originates from l-α-tyrosine. Initially, MdpC4 generates (*S*)-β-tyrosine from l-α-tyrosine. The product is then activated and tethered to a peptidyl carrier protein (PCP), MdpC2, by the stand-alone adenylation enzyme, MdpC1, to form (*S*)-β-tyrosyl-S-MdpC2 intermediate. The PLP-dependent transaminase, MdpC7, then eliminates the β-amino group followed by several modifications on the aromatic ring catalysed by hydroxylase, MdpC, *O*-methyltransferase, MdpC6, and halogenase, MdpC3. Stereospecific reduction of the β-carbonyl group is proposed to be catalysed by MdpC8, before attachment to the enediyne core by the condensation enzyme, MdpC5.^83^

### 2.5 Pradimicins

**Fig. 10:**
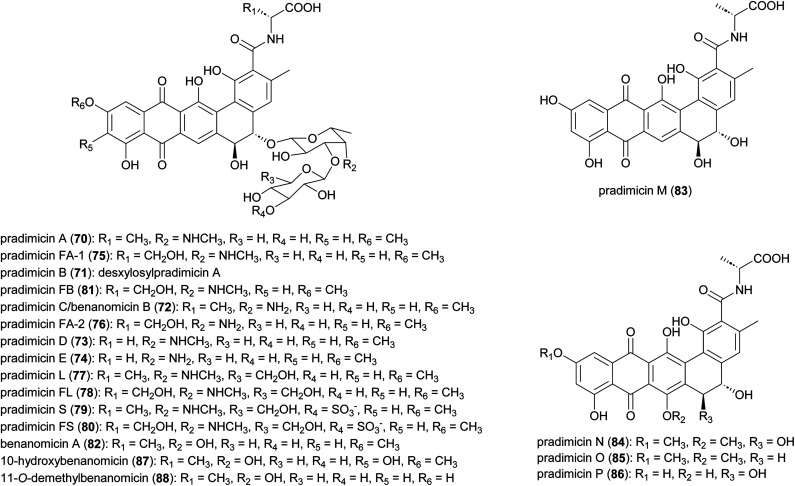
Structures of pradimicins.

Antifungal and antiviral compounds pradimicns A–E (70–74), FA-1 (75), FA-2 (76) isolated from *A. hibisca* P157-2 (ATCC 53557),^85–87^ pradimicns L (77) and FL (78) from *A. verrucosopora* subsp. *neohibisca* R103-3,^88^ pradimicns S (79), FS (80), FB (81) from *A. spinosa* AA0851,^89,90^ and benanomicin A (82) produced by *A. spadix* MH193-16F4,^91,92^ all share the common 5,6-dihydrobenzo[*α*]naphthacenequinone skeleton but have variations in the amino acid and disaccharide moieties (Fig. 10). These metabolites demonstrate potent *in vitro* growth inhibitory effects on various fungi and yeasts, and *in vivo* protective effects in mice infected with *C. albicans*, *Cryptococcus neoformans*, and *Aspergillus fumigatus*.^85,86,88,90,93–98^ Additionally, they exhibit potent activity against human immunodeficiency virus (HIV),^94,98,99^ and influenza virus.^93,94^ Pradimicns FA-1, FA-2, FB, FL, and FS, obtained by supplementing the culture medium with d-serine, leading to replacement of d-alanine in pradimicns A, C, B, L and S, respectively.^87,88,90^ Mutagenesis of *A. hibisca* P157-2 yielded a mutant strain that produced pradimicins D and E at much a higher titer.^86^ Other mutants were obtained, yielding deglycosylated metabolites pradimicins M–P (83–86), devoid of antifungal activity, emphasizing the importance of sugar moieties for activity.^100^ Benanomicin analogues 10-hydroxybenanomicin (87) and 11-*O*-demethylbenanomicin (88) were generated by biotransformation of benanomicin A using an *E. coli* BL21(DE3) strain co-overexpressing the cytochrome P_450_*cphP* from an unknown actinomycete with *Pseudomonas* redox partner genes *camAB*. Both analogues showed reduced antifungal activity compared to benanomicin A.^101^

The most intriguing feature of pradimicins is their d-mannopyranoside binding specificity in the presence of Ca^2+^ ions. These are the only non-peptidic natural compounds with lectin-like characteristics. Through this unique ability to bind to mannose-containing glycans, they inhibit the growth of pathogenic fungi, HIV, influenza virus, coronavirus, and *Trypanosoma brucei*.^93,102–107^ Investigations into the molecular basis of d-mannose binding of pradimicins led to their application in microbial cell surface imaging through coupling to a tetramethylrhodamine fluorophore.^108–112^

**Fig. 11:**
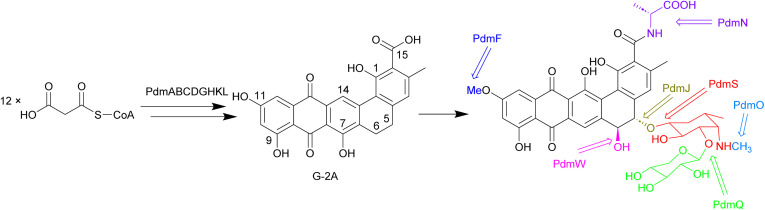
The biosynthesis of pradimicins is directed by the *pdm* gene cluster, with PdmABC acting as the minimal type II polyketide synthase enzymes. Modifications by ligases, P_450_ enzymes, methyltransferases, and glycosyltransferases lead to the final molecule.

Biosynthesis of the pradimicins is directed by the *pdm* gene cluster.^113^ The minimal type II polyketide synthase enzymes, PdmA, B and C, along with tailoring enzymes PdmD, G, H, K and L, were identified by heterologous expression and combinatorial biosynthesis to be involved in synthesis of the intermediate benzo[*α*]naphthacenequinone G-2A from 12 malonyl-CoA (Fig. 11).^114,115^ The amino acid ligase PdmN has relaxed substrate specificity and can introduce d-alanine or d-serine moieties at the C-15 position of G-2A.^114,116^ Two cytochrome P_450_ enzymes, PdmJ and PdmW, hydroxylate C-5 and C-6, respectively.^114,116,117^ Through gene deletion and enzyme studies, it was determined that the *O*-methyltransferase PmdF is accountable for the 11-*O*-methylation process. Additionally, PdmT was identified as a 7-*O*-methyltransferase, which is subsequently followed by a yet to be characterized process involving C-7 demethoxylation and C-14 hydroxylation.^118,119^ The glycosyltransferase PdmS is responsible for attachment of the first sugar, 4′,6′-dideoxy-4′-amino-d-galactose, at the 5-OH, followed by attachment of d-xylose to the 3′-OH of the first sugar, catalysed by PdmQ.^120,121^ The *N*-methyltransferase PdmO has been suggested to methylate the 4′-NH_2_ of the 4′,6′-dideoxy-4′-amino-d-galactose moiety.^121^

### 2.6 Angucyclines

**Fig. 12:**
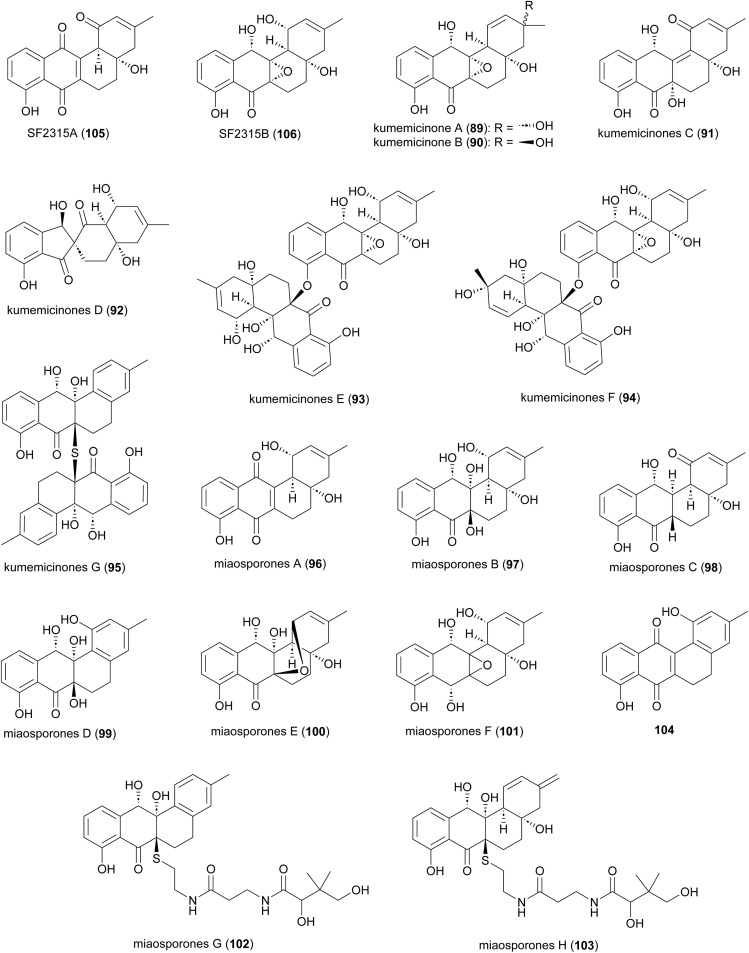
Structure of angucyclinones.

Several angucyclinone polyketides have been reported to be produced by *Actinomadura* strains: kumemicinones A–G (89–95) from deep-sea-derived *Actinomadura* strain KD439,^122^ miaosporones A–H (96–103) from *A. miaoliensis* TBRC 5172,^123^ and 5,6-dihydro-1,8-dihydroxy-3-methylbenz[*a*]anthracene-7,12-quinone (104) from marine *Actinomadura* strain DS-MS-114 (Fig. 12). These compounds are regioisomers of SF2315A (105) and SF2315B (106), which were also identified from culture broth of *A. miaoliensis* TBRC 5172 and *Actinomadura* strain KD439, respectively. These compounds tend to show generalised toxic effects. For example, 89–95 and 106 showed cytotoxic effects on P388 murine leukemia cells, and 96 showed cytotoxic against cancer cell lines MFC-F and NCI-H187, as well as nonmalignant (vero) cells.^122,123^96, 104, and 105 showed potent activity on the malaria parasite, *P. falciparum*, and the bacterial pathogen *Mycobacterium tuberculosis*.^123^104 exhibited activity against *S. aureus*.^124^ Compound 104 has also been patented for its immunosuppressant activity against Concanavalin A-induced blastogenesis of T lymphocytes,^125^ though it seems likely that this will also be based on general cell toxicity.

### 2.7 Anthrones

**Fig. 13:**
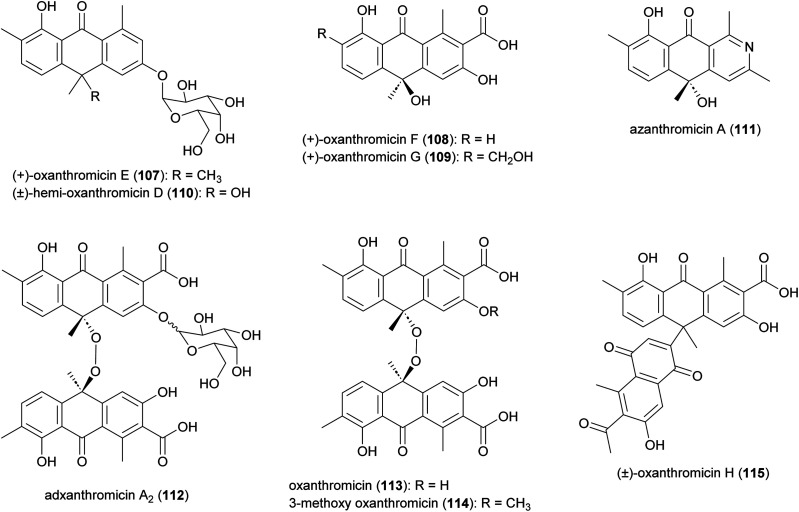
Structure of anthrones.

Anthrone derivatives (+)-oxanthromicins E–G (107–109), (±)-*hemi*-oxanthromicin D (110), azanthromicin A (111), adxanthromicin A_2_ (112), oxanthromicin (113), 3-methoxy oxanthromicin (114), and (±)-oxanthromicin H (115) were isolated from the *Actinomadura* strain BCC47066 (Fig. 13).^126^ Screening of these compounds against various pathogenic microorganisms and cancer cell lines revealed potent activity of adxanthromicin A_2_ against fungal plant pathogens *Colletotrichum capsica* and *Colletotrichum gloeosporioides* (MIC of 6.25 μg mL^−1^ for both) as well as antibacterial activity against *B. cereus* (MIC = 3.13 μg mL^−1^). In addition, (+)-oxanthromicin E demonstrated potent activity against *Herpes simplex* virus type 1 (IC_50_ = 2.33 μg mL^−1^).^126^ The dimeric anthrone peroxide oxanthromicin, first isolated from *Actinomadura* strain SCC 1646, showed broad-spectrum *in vitro* antifungal activity. The compound specifically exhibited good activity against dermatophytic fungi.^127,128^

### 2.8 Anthracyclines

**Fig. 14:**
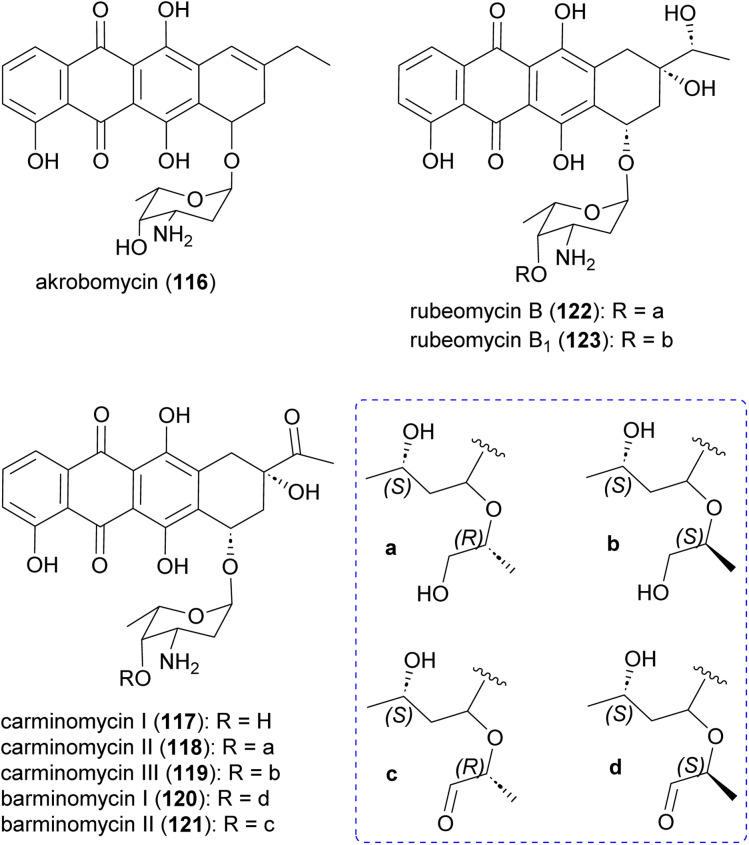
Structure of anthracyclines, DNA targeting compounds produced by *Actinomadura*.

Akrobomycin (116),^129^ carminomycins I (117),^130,131^ II (also known as rubeomycin A and 4-hydroxybaumycin A_2_) (118), and III (also known as rubeomycin A_1_ and 4-hydroxybaumycin A1) (119),^132–135^ barminomycins I (120) and II (121),^136,137^ and rubeomycins B (122) and B_1_ (123),^133,135^ are all produced by *Actinomadura* and are extremely potent cytotoxic agents (Fig. 14). Structurally they are closely related to the currently used anticancer drugs daunorubicin (daunomycin) and doxorubicin (adriamycin). Carminomycin I in a desmethyl analogue of daunorubicin with almost same antitumor activity. The cytotoxic activity of barminomycins I and II against P388 leukemia cells showed 1000-times higher potency than doxorubicin.^136^ The mode of action of barminomycins is unique because it can bind DNA in two different ways. One is through intercalation of the aglycone chromophore into DNA, and the second is through sequence-selective covalent binding to DNA after imine formation between aldehyde and amino sugar.^138–141^

### 2.9 Rubterolones, maduralactomycins and actinospirols

**Fig. 15:**
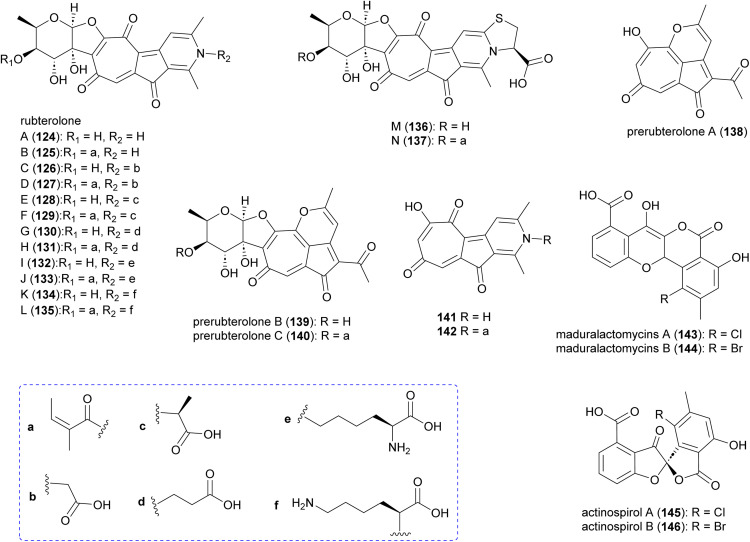
Structure of rubterolones, maduralactomycins and actinospirols.

Investigation of *Actinomadura* strain 5–2 (RB29), a bacterial symbiont of fungus-growing termites, revealed the polyketides rubterolones A–F (124–129), prerubterolones A–C (138–140), pyridine forms of prerubterolones A, 141 and 142, maduralactomycins A (143) and B (144), actinospirols A (145) and B (146), and lanthipeptides rubromicin A and B.^142–145^ Supplementation of culture media with β-alanine, l-lysine, and l-cysteine led to the production of additional rubterolone analogues G–N (130–137) (Fig. 15).^142,143^ Subsequently, several rubterolone congeners were produced by culture medium supplementation with synthetic primary amines.^143^ Chlorinated angucyclic compounds, the tetracyclic maduralactomycin A and the spirocyclic actinospirol A, were only produced when *Actinomadura* strain 5–2 (RB29) was grown from spores. Replacing NaCl with KBr in the culture medium gave rise to the brominated analogues 144 and 146.^144^ Labelling studies using ^13^C enriched acetate confirmed that maduralactomycins and actinospirols are produced by type II PKS enzymes and suggested the involvement of oxidative rearrangement, leading to two different scaffolds.^144^

**Fig. 16:**
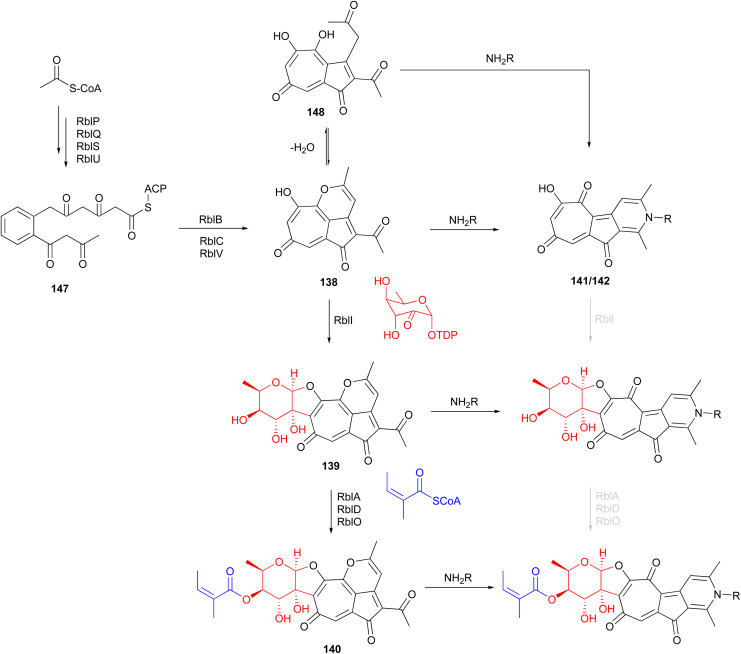
Proposed biosynthetic pathway for rubterolones involving a cascade of enzymes and nonenzymatic spontaneous pyridine ring formation from prerubterolones A–C (138–140).

The putative type II PKS gene cluster *rbl* was proposed to assemble the pentacyclic core of the rubterolones.^142^ A cascade of enzymes involving RblP (ACP), RblQ (chain length factor), RblS (KSII) and RblU (polyketide cyclase) are postulated to synthesize the intermediate 147 from malonyl-CoA starting units (Fig. 16). Intermediate 147 then undergoes oxidation, cyclization, rearrangement, and decarboxylation, catalysed by RblB, RblC, and RblV, respectively, to produce prerubterolone A, which is in equilibrium with its hydrolysed 1,5-dione form 148. Pyridine ring formation occurs spontaneously *via* the nonenzymatic capture of ammonium or a primary amine compound.^142^ In a culture medium with low NH_4_^+^ concentration, prerubterolones A–C (138–140) were isolated.^143^ The addition of glycine or NH_4_OH to an extract obtained from the microbial culture with low NH_4_^+^ concentration led to immediate pyridine ring formation, suggesting not only the spontaneous nature of pyridine formation but also that prerubterolones A–C are indeed the biosynthetic precursors of rubterolones.^143^ The O,C-condensation of dTDP-2-keto-6-deoxy-d-glucose is proposed to be catalysed by glycosyltransferase RblI. All genes encoding the enzymes necessary for the putative route to biosynthesis of dTDP-2-keto-6-deoxy-d-glucose have thus been identified in the *rbl* gene cluster.^142^ Based on the isolation of precursors 139 and 140, it is suggested that the glycosyltransferase RblI has relaxed substrate specificity and is capable of transforming both pyridine and pyran intermediates into glycosylated products prerubterolone B and rubterolone A (or other *N*-substituted congeners),^143^ but it is also possible that prerubterolone A is the only substrate for RblI to produce prerubterolone B and finally C as true natural products that are spontaneously transformed to pyridine form by capture of ammonium or a primary amine from the culture medium. The same scenario is plausible for the enzymes involved in attachment of coenzyme A activated (*Z*)-2-methyl-2-butenoic acid (angelic acid) to C-4 of the sugar.

Anti-inflammatory activity assays revealed that rubterolone D moderately decreases the prostaglandin E_2_ in humane monocytes, and also that prerubterolone C and rubterolone N inhibit the activity of microsomal prostaglandin E_2_ synthase 1, by 68% and 81%, respectively, relative to control.

### 2.10 Xanthones

**Fig. 17:**
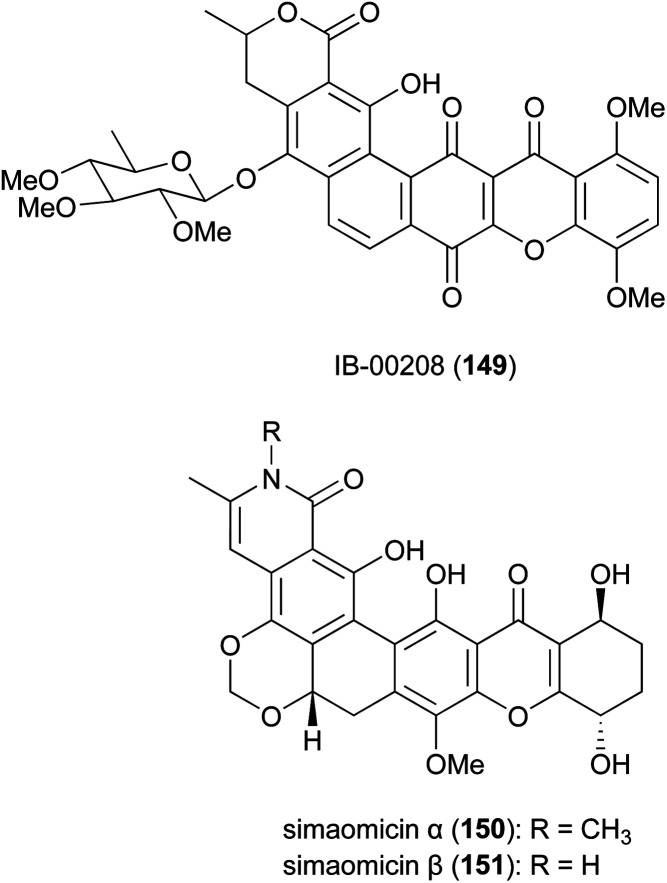
Xanthones from *Actinomadura*.

The polycyclic xanthone IB-00208 (149) (Fig. 17), isolated from the marine-derived *Actinomadura* strain BL-42-PO13-046, exhibited significant activity against several human and murine tumour cell lines, including P388D1, A-549, HT-29 and SK-MEL-28 at MICs of 1 nM.^146,147^ However, the compound is likely to be non-specifically (and highly) toxic, as it also acts as a potent growth inhibitor of Gram-positive bacteria such as *S. aureus, B. subtilis* and *M. luteus* (MICs in range of 0.09 to 1.4 nM). It did not show any activity against tested Gram-negative pathogens, presumably because it does not penetrate the outer membrane.^146^ Total synthesis of the aglycone of 149 has been accomplished in 22 steps. However, the final hexacyclic aglycone is an inseparable mixture of hydroquinone-quinone tautomers.^148^

Simaomicins (LL-D42067) α (150) and β (151) are derived from *A. madura*.^149,150^ Simaomicin α is highly potent against several Gram-positive bacteria (MIC = 0.06 μg mL^−1^), malaria parasite *P. falciparum*, and is a potent broad spectrum anticoccidial. Added to the diet of chickens, at an optimal dosage of 1 ppm, the compound almost completely prevented lesions caused by a variety of *Eimeria* species.^149–151^ In addition, simaomicin α also showed antiproliferative activity against various tumour cell lines with IC_50_ values in the range of 0.3–19 nM, *via* suppression of retinoblastoma protein phosphorylation, leading to cell cycle arrest in the G_1_ phase.^152^

### 2.11 Other polyketides

**Fig. 18:**
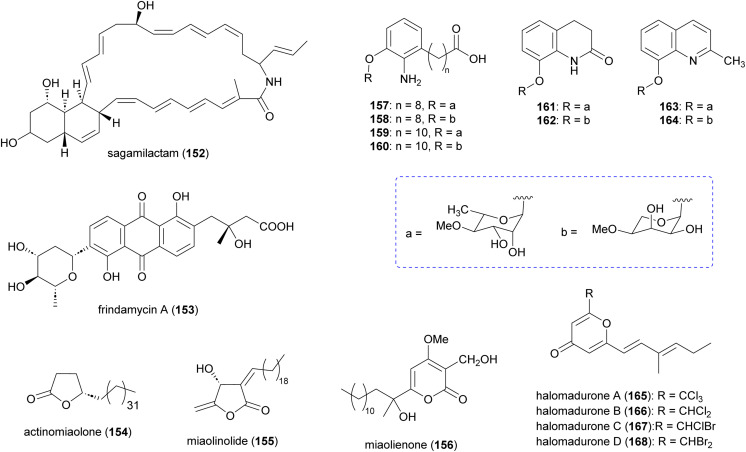
Other polyketides isolated from culture extract of *Actinomadura*.

In addition to the aforementioned polyketide natural products, *Actinomadura* spp. produce several other polyketides (Fig. 18). The 34-carbon membered macrocyclic lactam sagamilactam (152), isolated from *Actinomadura* strain K13-0306, showed potent growth inhibitory activity against *T. brucei* (IC_50_ value 0.25 μM).^153^*Actinomadura* strain RB99, isolated from the surface of a fungus-growing termite, produces the type II PKS-derived metabolite fridamycin A (153).^154^ This compound induces glucose uptake in 3T3-L1 cells through activation of an AMP-activated protein kinase signalling pathway. In contrast to the commonly used antidiabetic medicine rosiglitazone, it has the benefit of not affecting lipid accumulation and weight gain.^154^ The γ-lactone actinomiaolone (154), butanolide miaolinolide (155), and α-pyrone derivative miaolienone (156) were isolated from *A. miaoliensis* BCRC 16873.^155,156^ Miaolinolide and miaolienone strongly inhibited the production of lipopolysaccharide-induced tumor necrosis factor (TNF-α) in U937 cells *in vitro*, with IC_50_ values of 0.76 and 0.59 μM, respectively.^156^*Actinomadura* strain BCC27169 yielded a series of 3-oxyanthranilic acid derivatives (157–164). These derivatives were associated with three distinct aglycones, which were attached to either 4-*O*-methylrhamnose or 4-*O*-methylribopyranoside monosaccharides. Metabolites 159 and 164 showed activity against *M. tuberculosis* H37Ra at MIC of 50 μg mL^−1^. Furthermore, 157 exhibited cytotoxicity against human epidermoid carcinoma KB cells with IC_50_ of 19 μg mL^−1^.^157^

Chlorinated metabolites, halomadurones A (165) and B (166), are produced by *Actinomadura* strain WMMB499, isolated from the ascidian *Ecteinascidia turbinate*. Increasing the KBr/NaCl ratio led to production of halomadurones C and D. The brominated analogues, 167 and 168, were shown to be potent activators of NF-E2–related factor 2 (Nrf2), indicating that bromination is important for activity. The utility of these compounds is again unclear as they exhibit toxicity at higher concentrations.^158^

## 3. Nonribosomal peptides

### 3.1 Madurastatins

**Fig. 19:**
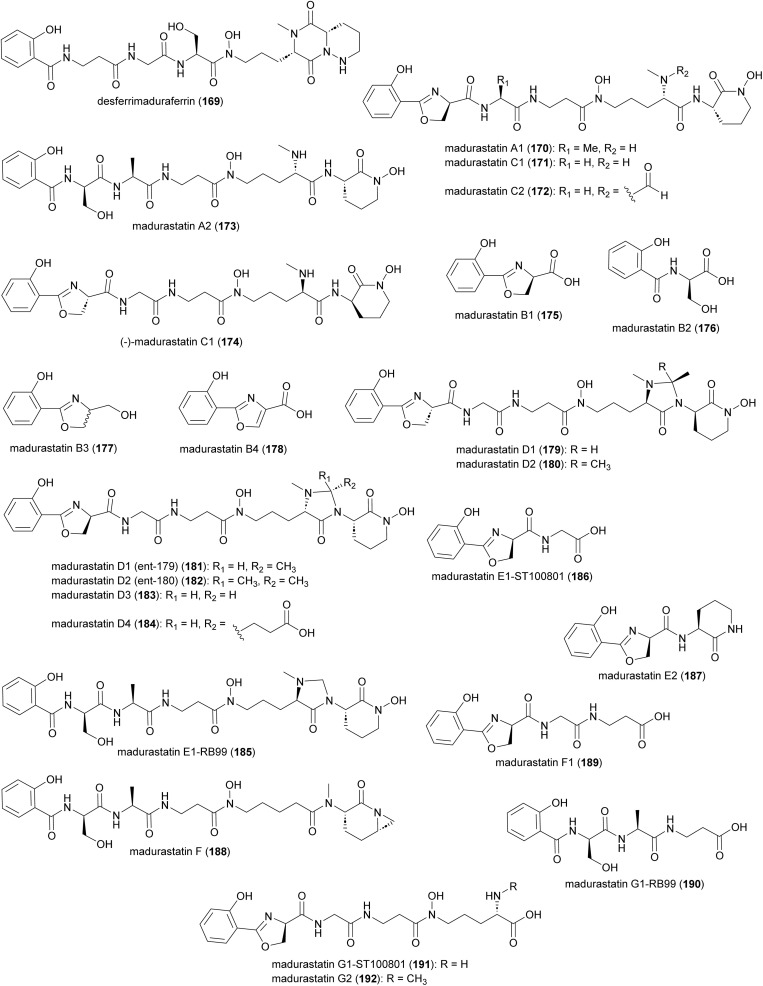
Structure of madurastatins isolated from *Actinomadura* strains.

Production of multiple phenolate-hydroxamate siderophores, including desferrimaduraferrin (169) and madurastatins (**170–192**), have been reported for *Actinomadura* spp (Fig. 19).^159–164^ Initially madurastatins were characterized as aziridine containing peptides.^160,161^ Revisiting the NMR data and comparison with siderophores having 2-(2-hydroxyphenyl)oxazoline moieties, together with partial synthesis, suggested the revision of the aziridine to an oxazoline ring.^165,166^ Compounds 185 and 186, despite having different structures, share the common name of Madurastatin E1. Likewise, compounds 190 and 191, despite their structural differences, are both referred to as madurastatin G1.^163,164^ To avoid confusion, we have renamed madurastatin E1 from *Actinomadura* strain RB99, E1-RB99 (185), and the molecule from *Actinomadura* strain ST100801 E1-ST100801 (186). Similarly, madurastatins G1 were renamed G1-RB99 (190) and G1-ST100801 (191). The absolute configuration of some madurastatins are still ambiguous. While the absolute configuration of madurastatin C1 has been assigned by Marfey's method, a congener named (−)-madurastatin C1, with identical analytical features but opposite optical rotation, has been identified.^162^ Similarly, enantiomers of madurastatins D1 (*ent*-179) and D2 (*ent*-180) has been reported.^164^ Some of the madurastatins possess moderate activity against *Micrococcus luteus*^162^ and *Moraxella catarrhalis*^164^ but that has been attributed to their iron-chelating mode of action.^164^

**Fig. 20:**
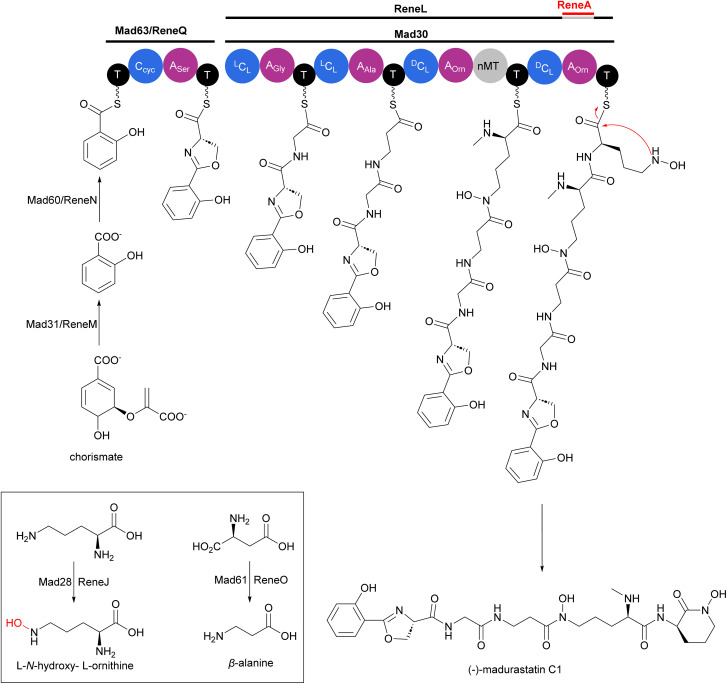
Biosynthesis of (−)-madurastatin C1 by the NRPS *mad* gene cluster from *Actinomadura* strain WMMA-1423. The cluster includes enzymes for salicylate synthesis. Homologous enzymes from the *rene* gene cluster are also shown.

Two independent studies proposed NRPS biosynthetic gene clusters *mad* and *rene* from *Actinomadura* strains WMMA-1423 and RB99, respectively, to be responsible for the biosynthesis of madurastatins.^162,163^ For simplicity, we will focus on the biosynthesis of (−)-madurastatin C1 by the *mad* gene cluster, while also noting the homologous enzymes from the *rene* gene cluster for comparison (Fig. 20). Mad28 (ReneJ) and Mad61 (ReneO) were identified as putative l-ornithine N-monooxygenase and aspartate 1-decarboxylase enzymes, involved in the generation of the L-*N*-hydroxy-l-ornithine and β-alanine moieties, respectively. The putative salicylate synthase Mad31 (ReneM) and salicylate-AMP ligase Mad60 (ReneN) are proposed to generate salicylate from chorismate and to load the thiolation domain of Med63 (ReneQ). One modular NRPS Med63 (ReneQ), carrying a heterocyclization domain, catalyses the condensation of serine to salicylate followed by cyclization to form an oxazoline ring. Four modules of Mad30 (ReneL) then extend the chain by attachment of glycine (alanine or serine), β-alanine, and two *N*-hydroxy-l-ornithines. In the case of (−)-madurastatin, the C1 stereochemistry of both *N*-hydroxy-l-ornithines is inverted by the dual epimerase/condensation activities of the ^D^C_L_ domains of modules three and four. The *N*-methyltransferase at module three is responsible for *N*-methylation of the α-amine group of the ornithine installed by that module. Following attachment of the second *N*-hydroxy ornithine in the last module, (−)-madurastatin C1 is released from the assembly line *via* intramolecular nucleophilic substitution and generation of an *N*-hydroxy lactam. None of the studies describing the *mad* or *rene* gene clusters have reported the presence of a TE domain in the last module. The enzyme involved in the release mechanism through intramolecular cyclization has yet to be identified. The adenylation domain is missing in the last module of the *rene* gene cluster of *Actinomadura* strain RB99. Instead, the gene encoding a transacting adenylation domain, ReneA, was identified in the cluster and suggested to be responsible for incorporation of last ornithine or *N*-hydroxy-l-ornithine in the peptide chain.^163^

### 3.2 GE23077

**Fig. 21:**
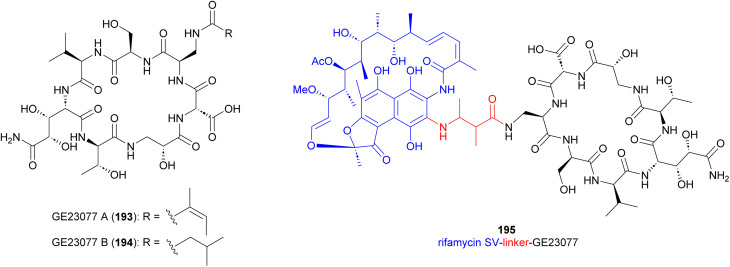
Structure of GE23077 A and B and GE23077 linked to rifamycin SV (195).

Cyclic peptide antibiotics GE23077 A (193) and B (194) (Fig. 21), produced by *Actinomadura* strains GE23077 and DSMZ 13491, were identified based on their inhibitory activity on *E. coli* RNA polymerase (RNAP) *in vitro*. The crystal structure of an RNAP-GE23077 A complex helped to assign the absolute configuration of the amino acid residues of GE23077 A, except for a dihydroxy-glutamine moiety.^167^ Despite their potent *in vitro* activity on RNAP, they showed narrow spectrum activity on bacterial cells tested, their activity being limited to *Moraxella catarrhalis* isolates in the range of 4–8 μg mL^−1^.^168,169^ A combination of biochemical, genetics and structural studies revealed that the GE23077 antibiotics block transcription initiation *via* binding to the RNAP active centre ‘*i*’ and ‘*i* + 1’ nucleotide binding sites thereby inhibiting the first nucleotide-addition step in transcription initiation.^167^ Due to the proximity of the binding sites of GE23077 and rifamycin, bipartite RNAP inhibitors like compound 195 were developed. These inhibitors, which occupy the binding sites of both parent molecules, demonstrated improved potency and showed reduced susceptibility to target-based resistance.^167,170^

**Fig. 22:**
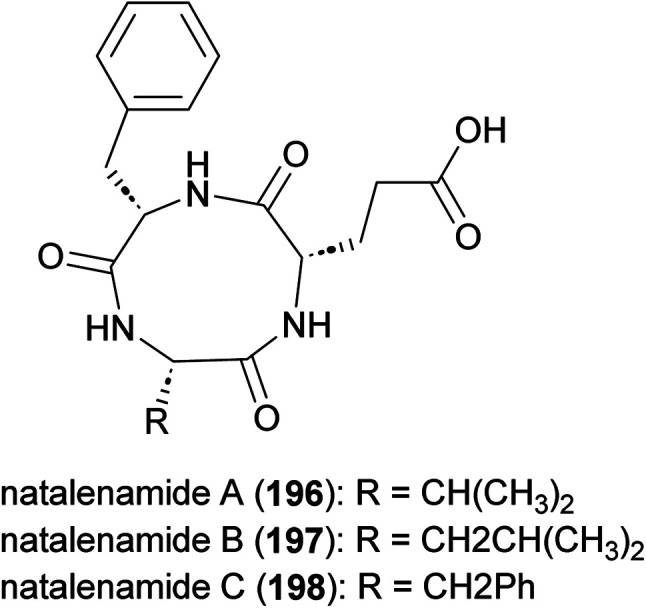
Structure of natalenamides.

Cyclic tripeptides natalenamides A–C (196–198) purified from the culture broth of *Actinomadura* strain RB99 isolated from fungus-growing termite (Fig. 22). Nantalenamide C showed to be a strong inhibitor of 3-isobutyl-1-methylxanthine-induced melanin synthesis, similar to the effect of kojic acid which is extensively used as skin-whitening agent in cosmetics. Nantalenamide A and B exhibited weak cytotoxicity against HepG2 and HeLa/A549 cancer cell lines.^171^

## 4. Hybrid polyketide-nonribosomal peptides

### 4.1 Matlystatins

**Fig. 23:**
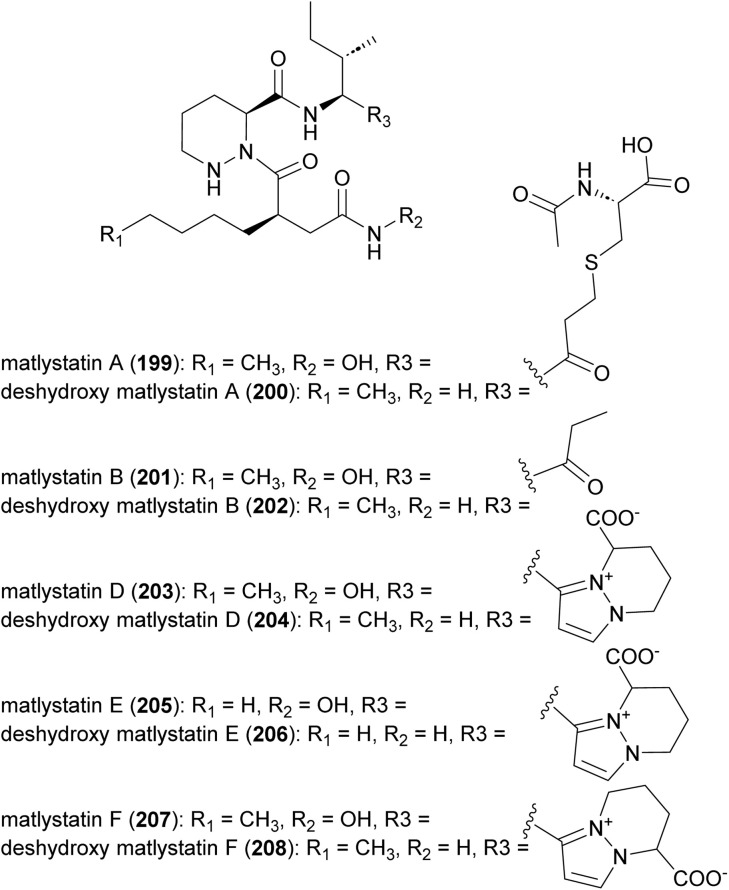
Structure of matlystatins.

Matlystatins isolated from *A. atramentaria* are potent inhibitors of type IV collagenases (Fig. 23).^172–174^ The compounds contain an *N*-hydroxy-2-pentyl-succinamic acid warhead, which is thought to inhibit the enzyme by binding to its active site zinc atom. The absolute configuration of matlystatin A, the most potent compound of the matlystatin series, was determined by total synthesis.^175^

**Fig. 24:**
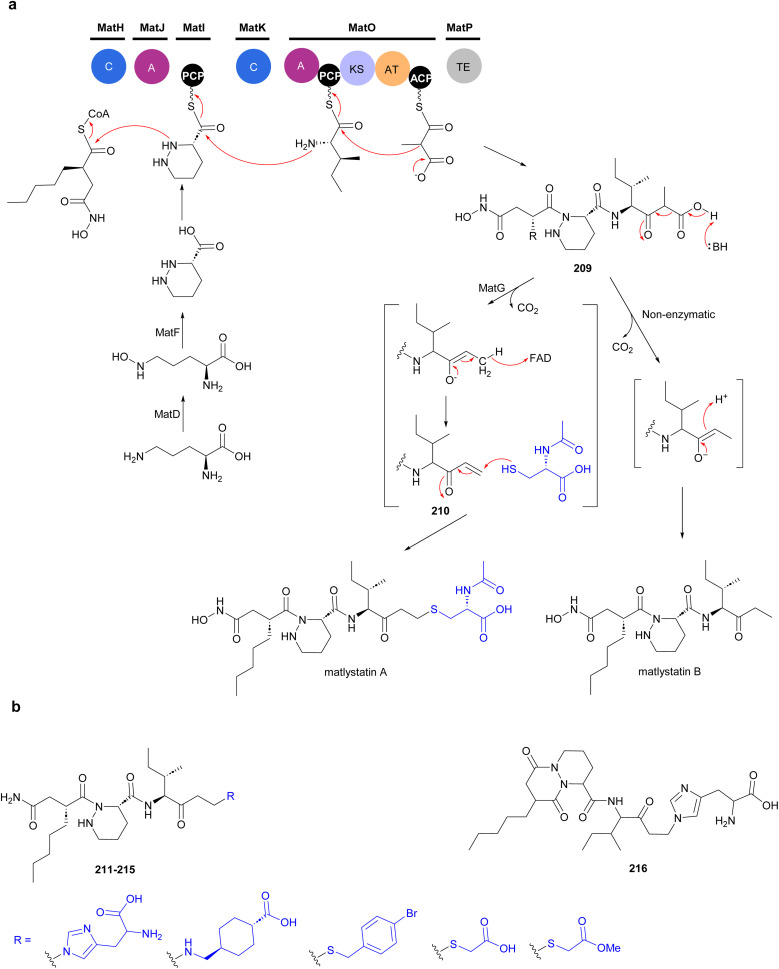
(a) Biosynthesis of matlystatins by a hybrid NRPS-PKS gene cluster. The unique decarboxylase–dehydrogenase MatG generates an electrophilic intermediate for the formation of carbon–sulfur or carbon–nitrogen bond in the final products. (b) Compounds produced by feeding different nucleophiles to culture medium.

The non-linear hybrid NRPS-PKS BGC, *mat*, that directs the biosynthesis of matlystatins was identified in the genome of *A. atramentaria* DSM 43919. A combination of heterologous expression, gene deletion and precursor feeding experiments were used to deduce the biosynthetic pathway (Fig. 24).^176^ The domains for two NRPS modules that assemble the psudotripeptide are encoded in five discrete genes, *matH*, *matI*, *matJ*, *matK* and *matO*, in the cluster. The *matO* gene also encodes a single PKS module that adds a methylmalonyl moiety to the tripeptide, which is then released by a standalone thioesterase MatP to give intermediate 209. Decarboxylation of this intermediate could happen either spontaneous or enzymatically. Non-enzymatic decarboxylation leads to the formation of matlystatin B. The most intriguing feature of matlystatin biosynthesis is the function of the decarboxylase–dehydrogenase, MatG, which generates the strongly electrophilic vinyl ketone intermediate 210. The vinyl ketone reacts with nucleophiles forming carbon–sulfur or carbon–nitrogen bonds in the final products, matlystatins A and D–F. Feeding other nucleophiles to the culture medium resulted in the production of several unnatural matlystatins 211–215. In addition, the matlystatin-like compound 216, with a fused bicyclic ring, was identified from a culture supplemented with histidine.^176^

### 4.2 Forazolines

**Fig. 25:**
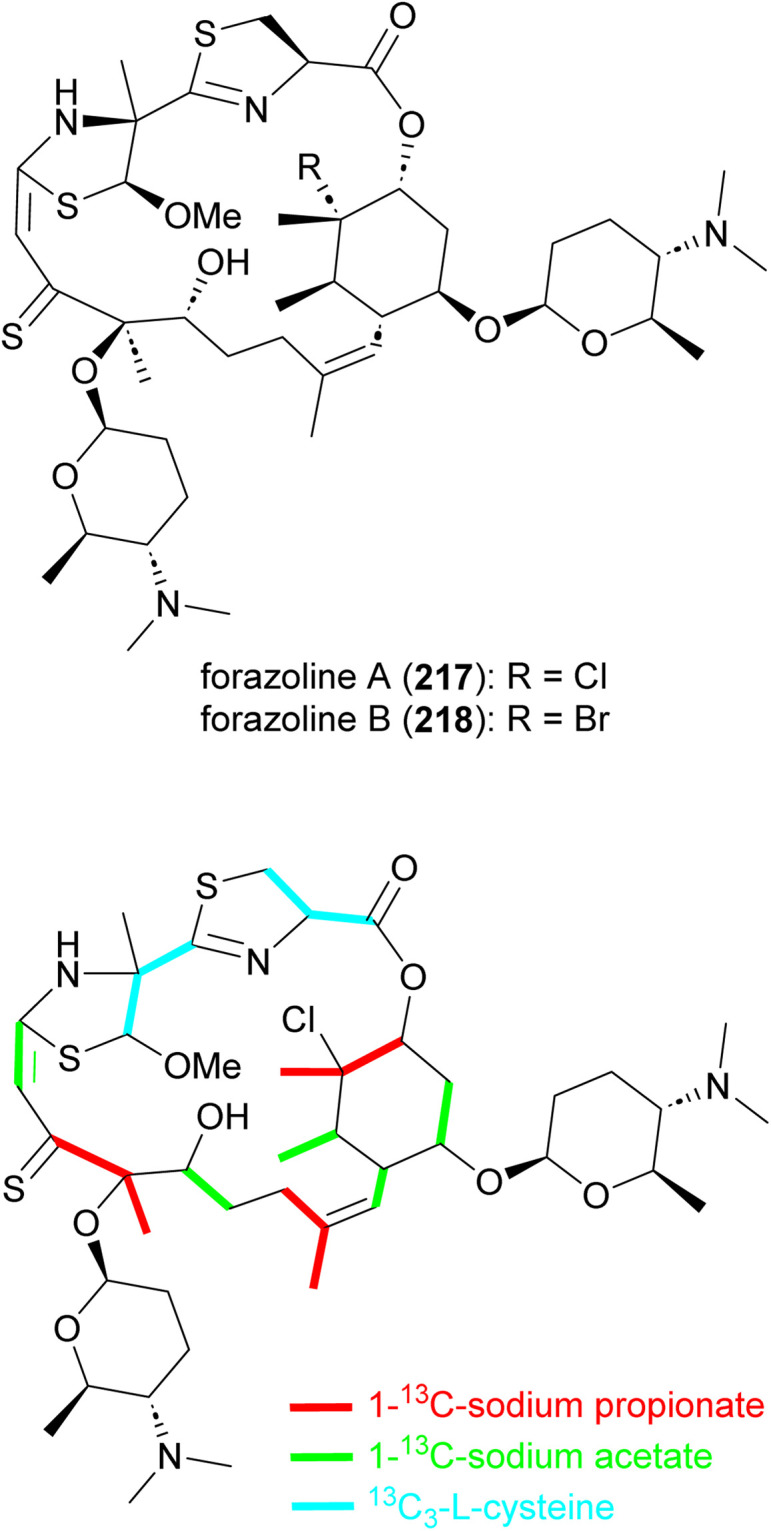
Structure of forazolines and incorporation of labelled propionate, acetate and l-cysteine into the structure of forazoline A.

Forazoline A (217), with an unusual thioketone group, is a chlorinated metabolite identified from the culture broth of *Actinomadura* strain WMMB499 (Fig. 25). The forazoline A structure initially reported had a sulfoxide group but the author later revised the structure based on the application of mass spectrometry-based isotopic fine structure and X-ray crystallographic data.^177,178^ Again, by taking advantage of the promiscuity of halogenases, an increased ratio of KBr/NaCl in the culture medium forced the production of brominated forazoline 218.^177^ Feeding experiments with ^13^C-labelled l-cysteine, sodium propionate, and sodium acetate suggested the incorporation of five units of malonyl-CoA, three methylmalonyl-CoA and two l-cysteines in the carbon backbone of forazoline by the hybrid NRPS-PKS machinery.^178^ The exact source of the PKS-derived thioketone is unknown. Both compounds (217 and 218) had *in vitro* activity against *C. albicans* with MIC values of 16 μg mL^−1^. Furthermore, *in vivo* studies of forazoline A revealed efficacy in a mouse infection model without toxicity. Chemical genomic experiments suggested a new mechanism of action based on the disruption of membrane integrity.^177^

**Fig. 26:**
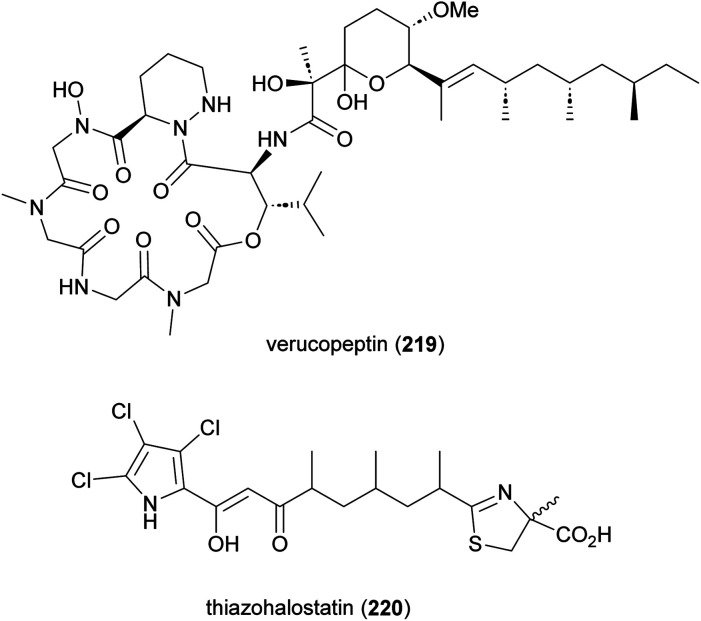
Structure of verucopeptin and thiazohalostatin.

Verucopeptin (219) and thiazohalostatin (220) are two other NRPS-PKS derived metabolites produced by *Actinomadura* spp (Fig. 26). The cyclodepsipeptide verucopeptin (219), initially identified from the culture broth of *A. verrucospora* Q886-3, showed potent cytotoxicity against various cancer cell lines and weak *in vivo* activity against mouse B16 melanoma.^179,180^ The compound was rediscovered in a screening assay targeting the transcription factor, hypoxia inducible factor-1 (HIF-1) and an absolute configuration was assigned.^181,182^ In further studies verucopeptin exhibited antitumor activity against multidrug resistant (MDR) cancer cells *via* a dual targeting mechanism involving dysregulation of v-ATPase and mTORC1 signalling.^183^ The BGC of verucopeptin was identified from *Actinomadura* strain XM-4-3, a mangrove rhizosphere bacterium.^184^ A set of verucopeptin analogues were made by genetic engineering and one-step semisynthesis, but none of these compounds had better activity than verucopeptin.^184^

In a screening program to find cytoprotective substances, thiazohalostatin (220) was identified from the culture broth of *Actinomadura* strain HQ24. Thiazohalostatin prevents cell death caused by calcium overload. The compound also showed inhibitory activity against lipid peroxidation.^185,186^

## 5. Miscellaneous

**Fig. 27:**
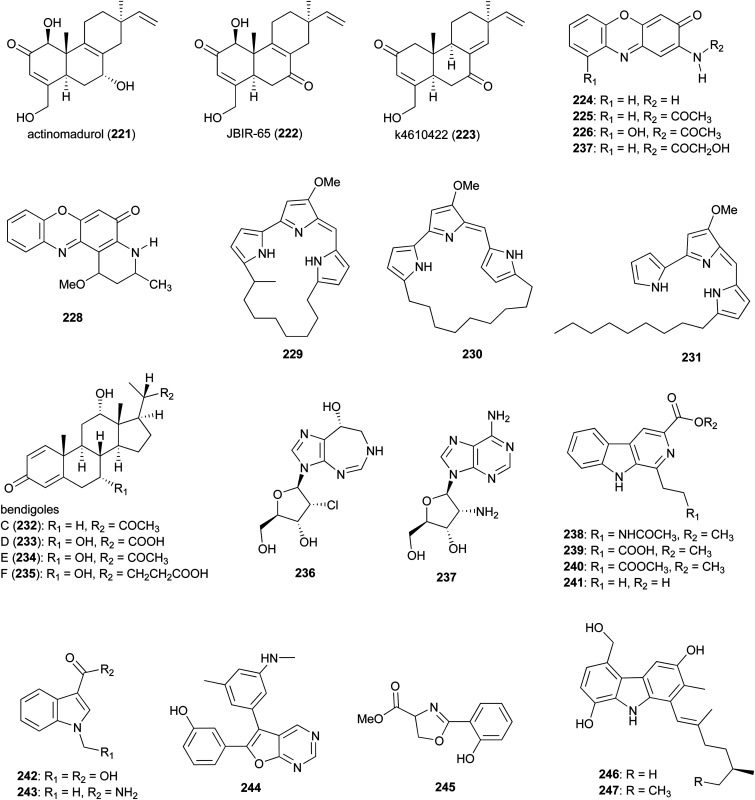
Miscellaneous metabolites produced by *Actinomadura*.

**Fig. 28:**
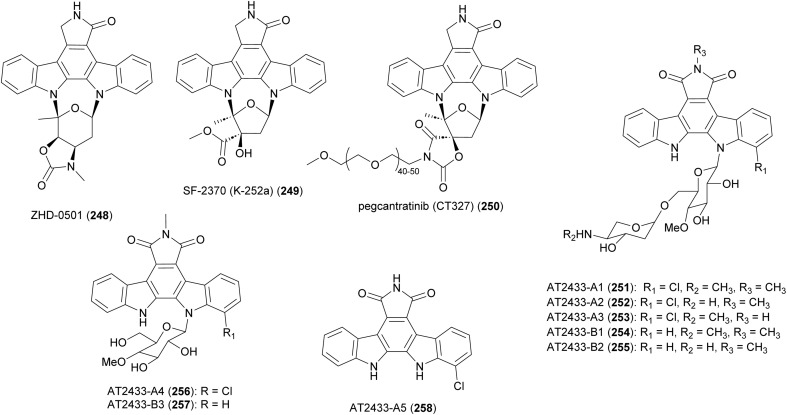
Structure of indolocarbazoles from *Actinomadura* strains and pegcantratinib (250) drug generated based on SF-2370 (K-252a).

While *Actinomadura* has been shown to be talented producer of polyketide natural products based on the compounds described above, only a few classes of compounds assembled by NRPS and hybrid NRPS-PKS biosynthetic machinery have been identified, so far, from culture extracts. In the case of ribosomally synthesised and post translationally modified peptides (RiPPs), only a few structurally related lantipeptides have been found. In addition to the above mentioned rubromicins A and B from *Actinomadura* strain 5-2 (RB29),^145^ the lantibiotic, cinnamycin B, was identified from culture extracts of *A. atramentria* NBRC 14695^T^, using a genome mining approach.^187^ Below, other miscellaneous secondary metabolites produced by *Actinomadura* strains are presented (Fig. 27 and 28).

Norditerpenoid metabolites actinomadurol (221) and JBIR-65 (222) were isolated from *Actinomadura* strain KC 191. While JBIR-65 had no detectable activity, the reduced form, actinomadurol, exhibited potent activity against pathogenic bacteria, *S. aureus*, *Kocuria rhizophila*, and *Proteus hauseri* with MICs in the range of 0.39–0.78 μg mL^−1^.^188^ JBIR-65 was isolated from sponge derived *Actinomadura* strain SpB081030SC-15. The compound found to protect neuronal hybridoma N18-RE-105 cells from l-glutamate toxicity (EC_50_ value of 31 μM).^189^ Another closely related norditerpenoid, k4610422 (223), with moderate cytotoxic activity against P388 leukemia cells, was purified from culture extracts of a thermophilic *Actinomadura* sp.^190^ Phenoxazinone derivatives questiomycin A (224), *N*-acetylquestiomycin A (225), and chandrananimycins A–C (226–228) were isolated from marine derived *Actinomadura* strain M048.^191^ These compounds were found to be active against various human cell lines. In addition, questiomycin A and chandrananimycin C exhibited strong antifungal activity against *Mucor miehei* and antialgal activity against the microalgae *Chlorella sorokiniana* and *C. vulgaris*.^191^

Cytotoxic prodiginines methylcyclooctylprodigiosin (229), cyclononylprodigiosin (230), and nonylprodigiosin (231) were isolated from *Actinomadura* strain BRA 177.^192^ Compounds 230 and 231 were originally reported from *A. madurae* and named cyclononylprodiginine and nonylprodiginine.^193,194^ Bioactivity guided isolation identified anti-inflammatory compounds bendigoles C–F (232–235) from the culture extract of marine derived *Actinomadura* strain SBMs009.^195^ Amongst them, bendigole F displayed the most inhibitory activity in an assay for translocation of NF-κB into the nucleus, with an IC_50_ of 71 μM. While bendigoles E and F were non-toxic, bendigole D showed moderate cytotoxicity against a mouse fibroblast L929 cell line with an IC_50_ of 30 μM.^195^

Adenosine-derived nucleoside antibiotics 2′-chloropentostatin (236), also known as adechlorin, and 2′-amino-2′-deoxyadenosine (237) are coproduced by *Actinomadura* strain ATCC 39365.^196–200^ 2′-chloropentostatin is a tight-binding inhibitor of adenosine deaminase with a *K*_*i*_ of 1.1 × 10^−10^,^197^ and 2′-amino-2′-deoxyadenosine exhibits potent *in vitro* and *in vivo* activity against a broad spectrum of DNA viruses and several RNA viruses.^201,202^ Both metabolites are suggested to be synthesised by the products of a single gene cluster, *ada*, albeit through separate pathways.^203^ Feeding experiments with and without [U–^14^C]adenosine demonstrated that adenosine is the direct precursor for the synthesis of both compounds.^196^ β-carbolines 238–241 and indoles 242 and 243 were isolated from *Actinomadura* strain BCC 24717, of which 1-ethyl-β-carboline-3-carboxylic acid (241) exhibited cytotoxicity against Vero cells (IC_50_ = 35.91 μg mL^−1^) and 1-methyl indole-3-carboxamide (243) had activity against *C. albicans* (IC_50_ = 42 μg mL^−1^).^204^

The furopyrimidine derivative, CCpI (244), produced by *Actinomadura* strain AL2, isolated from granite rock, exhibited a strong antibacterial activity against Gram-positive bacteria *B. subtilis* and *S. aureus* MTCC 740 (MIC = 2.0 and 4 μg mL^−1^, respectively), and moderate activity against Gram-negative bacteria *E. coli* and *Klebsiella pneumoniae* (MIC = 8.0 and 16 μg mL^−1^, respectively) but was not tested for toxicity. Based on *in silico* molecular docking studies it was suggested that CCpI could bind to various bacterial target molecules but the findings were not supported experimentally.^205^ Methyl-2-(2′-hydroxyphenyl)-2-oxazoline-4-carboxylate (245), isolated from a fermentation of *Actinomadura* strain MJ502-77F8, had moderate activity against Gram-positive bacteria, including different strains of *S. aureus* and *B. subtilis*.^206^ Carbazole alkaloids carbazomadurins A (246) and B (247), produced by *A. madurae* 2808-SV1, were identified during screening for the molecules protecting neuronal cells from glutamate toxicity. However, their mode of action was found to be linked to their antioxidative activity.^207^ The *S*-configured stereogenic center of carbazomadurin B was assigned by total synthesis.^208^ Total synthesis of carbazomadurin A was also achieved *via* two different approaches.^209,210^

Bacterial indolocarbazoles share a common core of indolo[2,3-*a*]pyrrolo[3,4-*c*]carbazole but subdivided into two groups of staurosporine and rebeccamycin-type indolocarbazoles based on their structure and mode of action.^211,212^ The staurosporine analogue, ZHD-0501 (248), was isolated from a fermentation broth of marine-derived *Actinomadura* strain 007 *via* bioactivity-guided separation procedure.^213^ ZHD-0501 exhibited strong antiproliferation activity against human cancer cell lines A549, HL60, BEL-7402, and mouse leukemia P388 cells, with inhibition rates of 83, 76, 57, and 62% at 1 μM, respectively. The compound also inhibited proliferation of tsFT210 mouse cancer cells but at lower rate. Flow cytometric analysis suggested that proliferation was being inhibited at the G2/M cell cycle phase.^213^ Total synthesis of ZHD-0501, achieved in 22 steps from d/l-glucose and 2,3-dibromomaleimide, confirmed the stereochemistry that was suggested based on coupling constants and NOE correlations.^214^ The synthesised ZHD-0501 along with several stereoisomers showed significant inhibitory activity against protein kinase C alpha/beta (PKC-α/β). ZHD-0501 inhibited PKC-α and PKC-β at IC_50_'s of 1.63 and 3.59 μM, respectively.^214^

Indolocarbazole SF-2370 (249) isolated from the culture broth of *Actinomadura* strain SF-2370 had moderate activity (MIC 6.25 μg mL^−1^) against a narrow spectrum of bacteria, specifically *Micrococcus luteus*, *Micrococcus flavus* FDA16 and *Corynebacterium bovis* 1810. It also showed a protective effect against rice plant diseases caused by *Pyricularia oryzae* (87% at 400 ppm), *Rhizoctonia solani* (86% at 200 ppm), and *Xanthomonas compestris* pv. *oryzae* (96% at 12.5 ppm).^215^ Almost at the same time, the compound was also identified from the culture broth of *Nocardiopsis* strain K-252a (named K-252a) as a potent inhibitor of protein kinase C (IC_50_ value of 32.9 nM).^216,217^ Subsequent studies revealed that the compound inhibits several other protein kinases.^218,219^ A PEGylated form of SF-2370 (K-252a), pagcantratinib (CT327) (250), was developed as inhibitor of tropomyosin-receptor kinase A (TrkA), which is associated with pruritus and psoriatic plaque formation. Conjugation of SF-2370 (K-252a) to PEG polymer improved the kinase inhibition as well as allowing for highly localized skin penetration, thus avoiding systemic adsorption and toxicity. Pagcantratinib is in phase IIb clinical trials for treatment of pruritus associated with psoriasis.^220^ Pegcantratinib has also been in a Phase II combination study with calcipotriene (vitamin D receptor agonist) to treat both pruritus and psoriasis.^221^

Antitumor antibiotics AT2433-A1 (251), A2 (252), A3 (253), A4 (256), A5 (258) and AT2433-B1 (254), B2 (255), B3 (257) are produced by *A. melliaura* ATCC 39691 (SCC 1655).^222–224^ Indolocarbazoles AT2433s are structurally close to rebeccamycin, both are β-*N*-glucosylated, but distinguished by their *N*-methylated and asymmetrically halogenated aglycone. In addition, the major compounds (A1–A3, B1 and B2) possess aminodideoxypentose containing disaccharides. Rebeccamycin is a potent inhibitor of topoisomerase I that works by stabilising the enzyme-DNA covalent complex.^225^ Addition of aminodideoxypentose to AT2433 enhances DNA affinity, but abolishes topoisomerase I inhibition.^226,227^ The terminal amino sugar was found to be important for both the DNA binding and putative topoisomerase-independent mode of action.^226,228^ A structure activity relationship study revealed the crucial role of the sugar moiety in their cytotoxicity. Disaccharide substituted AT2433 analogues 251–255 are more potent compared to monosaccharide substituted metabolites AT2433-A4 (256) and AT2433-B3 (257), and the monosaccharide conjugates are more active than the indolocarbazole aglycone AT2433-A5 (258).^224^ Similarly, it was found that chlorinated indolocarbazole AT2433s have higher antibacterial activity.^224^

**Fig. 29:**
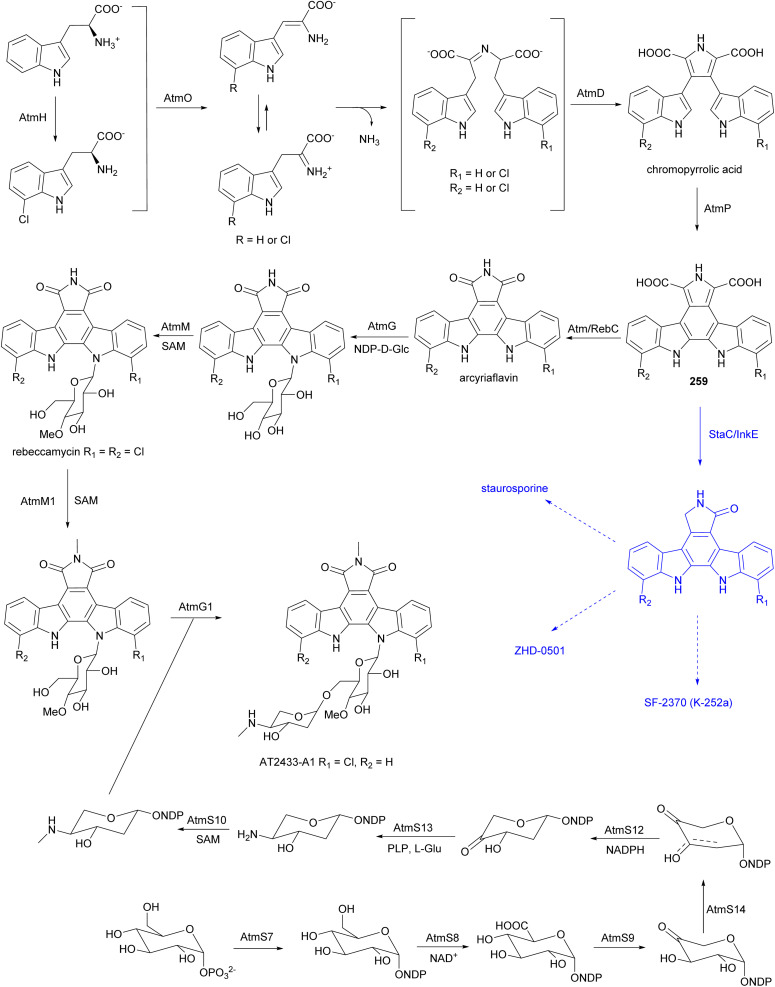
Proposed biosynthetic assembly of indolocarbazoles. Biosynthesis of two groups of indolocarbazoles bifurcate from intermediate 259. Substitution of RebC with StaC can switch the downstream products.

The biosynthetic gene cluster responsible for the AT2433 metabolites, *atm*, was identified by comparative genomics of the producer strain with the gene cluster of rebeccamycin and aminodideoxypentose-bearing enediyne calicheamicin, followed by confirmation through *in vitro* biochemical assays, as well as heterologous expression and *in vivo* bioconversion.^229–233^ Mainly based on the homology to the enzymes involved in the biosynthesis of rebeccamycin,^229–231,234–238^ the following biosynthetic pathway was proposed for the aglycone of AT2433 (Fig. 29). The flavin-dependent halogenase, AtmH, converts tryptophan to 7-chloro-l-tryptophan in the early steps of the biosynthesis. Both tryptophan and 7-chloro-l-tryptophan are substrates of the flavin-dependent l-amino acid oxidase, AtmO, to produce (7-chloro)indole iminopyruvate. The iminopyruvate can isomerise to the enamine, which acts as a carbon nucleophile on the imino carbon of the (7-chloro)indole iminopyruvate, leading to a dimerization event creating the intermediates that are coupled together by heme-dependent oxidase AtmD to form chromopyrrolic acid. The cytochrome P_450_ AtmP, and flavin-dependent monooxygenase AtmC (a homologue of RebC), together convert chromopyrrolic acid to arcyriaflavin through intermediate 259. The main divergence of the biosynthetic pathways of the aglycones of rebeccamycin and AT2433 *versus* staurosporine, ZHD-0501, and SF-2370 (K-252a), is the function of Reb/AtmC *versus* StaC/InkE (InkE is the Reb/Atm/StaC homologue in SF-2370 (K-252a) biosynthesis).^233,239–242^*In vitro* and *in vivo* experiments have shown that the products can be switched *via* substitutions of RebC and StaC.^239,240^

The *N*-glucosyltransferase AtmG installs glucose on the indolocarbazole aglycone and two SAM dependent *O*- and *N*-methyltransferases AtmM and AtmM1 are proposed as Glu-4′-*O*-methylase and maleimide *N*-methylase, respectively. The function of the glycosyltransferase AtmG and methyltransferases AtmM and AtmM1 in the biosynthesis of AT2433 have been verified biochemically.^233^ A cascade of genes involved in the biosynthesis of aminodideoxypentose was identified within the *atm* gene cluster, and the functions of several of these genes have been characterized.^233,243,244^ Biosynthesis of aminodideoxypentose is initiated by the α-d-glucose-1-phosphate thymidyltransferase AtmS7, then the TDP-α-d-glucose dehydrogenase AtmS8, and the TDP-glucuronic acid decarboxylase AtmS9, catalyse C-6 oxidative decarboxylation. Both the TDP-4-keto-α-d-xylose 2,3-dehydratase, AtmS14, and the TDP-2-deoxy-4-keto-α-d-pentos-2-ene 2,3-reductase, AtmS12, are required for C-2 deoxygenation. Subsequently, the TDP-2-deoxy-4-keto-β-l-xylose 3-aminotransferase, AtmS13, is postulated to catalyse amine instillation at the C-4 position. Finally, the methyltransferase AtmS10, *N*-methylates the aminopentose product before attachment to the aglycone catalysed by the *O*-glycosyltransferase AtmG1.

## 6. Conclusions

After a fruitful era of the discovery of specialized metabolites produced by Actinobacteria that have served as a source of life saving medicines, recent exploration of culture extracts of this prolific group of bacteria have mostly resulted in the rediscovery of known metabolites. Nevertheless, the emergence of MDR pathogens and the failure to obtain novel antibiotics from well-studied genus of Actinobacteria to tackle this issue, has led to a shift in focus, amongst other approaches, towards understudied genera of Actinobacteria, such as *Actinomadura*.

Various classes of natural products are produced by *Actinomadura* strains, but the majority of the compounds identified from their culture extracts are synthesised by polyketide synthase systems. The nonribosomal peptides and hybrid polyketide-nonribosomal peptide metabolites produced by *Actinomadura* strains appear to be specific to this genus. A wide range of metabolites have been reported and demonstrated to have diverse and sometimes unique activities. As described above, broad-spectrum antifungal antiviral metabolites pradimicins/benanomicins are unique non-peptidic carbohydrate binders. The anti-coccidian polyether maduramicin has the biggest market amongst all anti-coccidiosis agents. Indolocarbazoles produced by *Actinomadura* are potent protein kinase inhibitors and certain anthracyclines from this genus are extremely potent antitumour compounds. The potent antitumour agent, verucopeptin, with activity against MDR cancer cells, has a unique dual target mode of action which involves dysregulation of v-ATPase and mTORC1 signalling.

Several *Actinomadura* strains including *A. madurae*, *A. pelletieri*, and *A. latina*, have been identified as human pathogens and are frequently isolated from the infected tissues of actinomycetoma patients. However, their role in pathogenesis is not yet well understood.^10^ Bioinformatics analysis of the sequenced genomes of several of these isolates^245^ indicates that their genomes each contain more than 25 biosynthetic gene clusters, most of which are so far predicted to produce unknown metabolites. Understanding the secondary metabolites produced by these organisms could provide insights into their potential role in the actinomycetoma disease process.

Overall, *Actinomadura* represents a promising source of natural products that could be screened for activity against novel drug targets. By increasing our understanding of the biosynthesis of these compounds and their bioactivities, we can gain insights into their potential therapeutic uses and contribute to the discovery of new drug entities.

## 7. Conflicts of interest

There is no conflicts to declare.

## Supplementary Material
